# Nutritional Modulation of Immune Function: Analysis of Evidence, Mechanisms, and Clinical Relevance

**DOI:** 10.3389/fimmu.2018.03160

**Published:** 2019-01-15

**Authors:** Dayong Wu, Erin D. Lewis, Munyong Pae, Simin Nikbin Meydani

**Affiliations:** ^1^Nutritional Immunology Laboratory, Jean Mayer USDA Human Nutrition Research Center on Aging at Tufts University, Boston, MA, United States; ^2^Department of Food and Nutrition, Chungbuk National University, Cheongju, South Korea

**Keywords:** immune system, vitamin D, vitamin E, n-3 PUFA, probiotics, green EGCG, zinc

## Abstract

It is well-established that the nutritional deficiency or inadequacy can impair immune functions. Growing evidence suggests that for certain nutrients increased intake above currently recommended levels may help optimize immune functions including improving defense function and thus resistance to infection, while maintaining tolerance. This review will examine the data representing the research on prominent intervention agents n-3 polyunsaturated fatty acids (PUFA), micronutrients (zinc, vitamins D and E), and functional foods including probiotics and tea components for their immunological effects, working mechanisms, and clinical relevance. Many of these nutritive and non-nutritive food components are related in their functions to maintain or improve immune function including inhibition of pro-inflammatory mediators, promotion of anti-inflammatory functions, modulation of cell-mediated immunity, alteration of antigen-presenting cell functions, and communication between the innate and adaptive immune systems. Both animal and human studies present promising findings suggesting a clinical benefit of vitamin D, n-3 PUFA, and green tea catechin EGCG in autoimmune and inflammatory disorders, and vitamin D, vitamin E, zinc, and probiotics in reduction of infection. However, many studies report divergent and discrepant results/conclusions due to various factors. Chief among them, and thus call for attention, includes more standardized trial designs, better characterized populations, greater consideration for the intervention doses used, and more meaningful outcome measurements chosen.

## Introduction

The main functions of body's immune system are to protect the host against infection from pathological microorganisms, to clear damaged tissues, and to provide constant surveillance of malignant cells that grow within the body. Additionally, the immune system develops appropriate tolerance to avoid unwanted response to healthy tissues of self or harmless foreign substances. There is considerable heterogeneity among individuals in the vigor of their immunological function, largely owing to factors such as genetics, environment, lifestyle, nutrition, and the interaction of these factors. Nutrition as a modifiable factor in impacting immune function has been studied for several decades, and the research in this field has developed into a distinguished study subject called nutritional immunology. As with other bodily systems, the immune system depends on adequate nutrients to function properly. It is well-documented that nutritional status is closely associated with immunity and host resistance to infection. There is little argument that deficiency in both macronutrients and micronutrients causes immune function impairment, which can be reversed by nutrient repletion. Nutritional deficiencies are still prevalent in less developed regions and are a main contributor to a high incidence of morbidity and mortality from infectious diseases. Even in developed countries where general nutritional deficiencies are rare, nutrition issues such as specific nutrient deficiencies, less ideal diet composition, and excess calorie consumption are still a challenging reality. This situation is particularly significant in the elderly population due to a variety of factors more common in this population including disability, disease, disease-associated and medicine-induced anorexia, poor food selection, and lower socio-economic status. In addition, the aged may have greater requirements for certain dietary components to compensate for the deficit in cellular functions and increased stress associated with aging. While it is agreed that nutritional deficiency or insufficiency needs to be corrected to ensure that the immune system functions properly, mounting evidence suggests that for certain nutrients, increased intake above currently recommended levels may help optimize immune function including improving defense function and thus resistance to infection, while maintaining tolerance. Aside the known nutrients, there are a wide variety of non-nutritive phytochemicals and functional foods. They are not essential for maintaining normal cell metabolism and function thus do not have recommended levels of intake in dietary guidelines. Despite this, many phytochemicals and functional foods have been shown to have beneficial effects on immune function. This review will examine the data representing the research on prominent intervention agents (dietary lipids such as n-3 polyunsaturated fatty acids or PUFA), micronutrients (zinc, vitamins D and E), and functional foods (probiotics, tea components) for their immunological effect, working mechanisms, and clinical relevance. The intention of this review is to provide an updated overview on several prominent immuno-modulating food components, including the reported effects and modes of action, and current and potential clinical application. While there are many other members in each of above-mentioned categories that are also known to affect immune function, we have included only a few as representatives in the current review mainly based on the fact that they are relatively more intensively studied and their immuno-modulating properties are widely accepted although it is clearly acknowledged that discrepancy is far from resolved for the nature and magnitude of their actions, as well as in the efficacy and translational value of their potential application.

## Modulation of Immune Function by Nutrients and Food Components

In all the bodily systems and tissues, appropriate supply of different types of nutrients is essential for maintaining cell homeostasis and performing respective functions. While the immune system is no exception, its specific defense functions determine that immune cells may be particularly sensitive to the status of certain nutrients and food components. A primary task in nutritional immunology research is to identify such dietary factors and to define their optimal intake in terms of maintaining immunological balance and strengthening defense against pathogens.

### Vitamin D

Vitamin D is unique compared to other vitamins in that human body can synthesize it in the skin from the precursor 7-dehydrocholesterol when exposed to sunlight. Both sunlight-induced and diet-derived vitamin D are first hydroxylated to 25(OH)D mainly in liver, and further hydroxylated, under action of 1-α-hydroxylase, to the active form 1,25(OH)_2_D mainly in kidney. The classical function of vitamin D has long been recognized to be the regulation of calcium homeostasis and bone health. However, more extra-skeletal effects of vitamin D have been revealed, and the diverse functions of vitamin D are also supported by the discovery that vitamin D receptor (VDR) and vitamin D-activating enzymes (hydroxylases) are present in the tissues and cells not involved in mineral and bone metabolism.

#### Immunologic Effect and Mechanism

The extra-skeletal effects of vitamin D are well exemplified in the immune system. Most immune cells express VDR and some of them can produce 1-α-hydroxylase; in this way, both systemic and locally generated vitamin D in its active form can act on VDR expressed by immune cells in endocrine, paracrine, and autocrine manners. Indeed, vitamin D has been shown to broadly impact functions of immune cells in both the innate and adaptive immune system, as well as the antigen-presenting cells (APC) that links the two arms of immunity.

While vitamin D has been shown to influence different innate immune cells as well as the different functions of a given type of cells in varied manners, the overall effect of vitamin D on the innate immunity is stimulatory. Effects of vitamin D on monocytes and macrophages are recognized the earliest and also most intensively studied [reviewed in (1, 2)]. Human monocytes can be stimulated to proliferate when incubated in the presence of 1,25(OH) _2_D3 at physiological concentrations (3). In addition, 1,25(OH) _2_D3 promotes the chemotactic and phagocytic capacity of macrophages (4). Furthermore, 1, 25(OH) _2_D3 can induce production of several endogenous antimicrobial peptides in monocytes, neutrophils, and epithelial cells, such as cathelicidin and defensins (5–7). Together, vitamin D by stimulating all these innate antimicrobial immune responses can enhance elimination of invading bacteria, viruses, and fungi.

Vitamin D can also significantly influence the adaptive immune response. VDR and vitamin D-activating enzymes are found in both T and B cells (8). Activation of T or B cells, and their subsequent proliferation, can greatly elevate expression of VDR from low basal levels at rest. In contrast to its effect on the innate immunity, vitamin D is in general inhibitory on both T and B cells (9). In T cells, vitamin D inhibits T cell proliferation (10), and effector functions of both CD4^+^ and CD8^+^ T cells (11, 12). In particular, vitamin D inhibits production of IL-2 and IFN-γ, two key T cell cytokines (13). This is believed to be mediated through 1,25(OH) _2_D3-VDR dimerization with the partner nuclear receptor retinoid X receptor to form a functional VDR DNA-binding domain, which induces repression of several transcription factors regulating gene activation of IL-2 (14) and IFN-γ (15). Vitamin D can also impact T cell function by modulating CD4^+^ T cell differentiation into subpopulations. Naïve CD4^+^ T cells (Th0) can differentiate into different effector subsets, such as Th1, Th2, Th17, and regulatory T (Treg) cells after TCR engagement and co-stimulation in the presence of specific cytokines produced by the innate immune system upon encountering particular pathogens. Th1 and Th17 cells are involved mainly in immunity against intracellular pathogens, while Th2 cells are responsible for humoral immunity and targeting extracellular pathogens. Treg cells assist in the maintenance of self-tolerance and regulate immune responses to prevent excessive and mis-directed actions. Th1 and Th17 are thought to promote inflammation and autoimmunity, whereas Th2 and Treg are believed to have the opposite role. Although controversy exists, overall it appears that vitamin D restricts CD4^+^ T cell polarization toward the pro-inflammatory Th1 and Th17 cells while favoring the regulatory Th2 and Treg cell development (1, 12, 16).

Vitamin D has also been shown to affect APC function, primarily dendritic cells (DC). DC play an important role in controlling the development of adaptive immunity by appropriately conveying Ag signals to T cells. It is believed that some effects of vitamin D on adaptive immune response are mediated through DC (17). Vitamin D inhibits not only DC differentiation from their bone marrow and monocytic precursor cells, but also their maturation (18). A general consensus is that vitamin D helps program DC for tolerance and this feature affords vitamin D a therapeutic potential application in the clinic to alleviate autoimmune and inflammatory diseases.

#### Clinical Relevance

Given the effects of vitamin D on different aspects of immune functions mentioned above, adequate intake of vitamin D is anticipated to help maintain/strengthen the body's defense against infection by promoting the innate immunity. Conversely, its regulatory effect on T cells and DC suggest that vitamin D may help mitigate T cell-mediated autoimmune inflammatory diseases. Although the clinical studies have demonstrated some promising effects of vitamin D supplementation on several infection outcomes including tuberculosis, upper respiratory tract infection, hepatitis C virus, and HIV, the presence of great discrepancy among studies disallows for a definitive conclusion (19–21). Similarly, the evidence for the protective effect of vitamin D on autoimmune diseases does not seem to be consistent either. Some animal studies have shown that vitamin D supplementation is effective in preventing or alleviating inflammatory bowel disease (IBD), multiple sclerosis (MA), rheumatoid arthritis (RA), systemic lupus erythematosus, and Type 1 diabetes (T1D) in animal models (22, 23). Yet in humans, while epidemiologic studies have shown association between low vitamin D levels and incidence/severity of certain autoimmune diseases, the interventional trials have thus far generated inconsistent results (24, 25).

### Vitamin E

Vitamin E is a generic term for all tocopherols and tocotrienols that exhibit the biological activity of α-tocopherol. Although α- and γ-tocopherols, the main forms of vitamin E, are similarly abundant in the diet, α-tocopherol is about 5 to 10-fold higher than γ-tocopherol in blood due to the different preference in bioavailability and metabolism. All the other forms of vitamin E are very low or undetectable in the body tissues. Both synthetic and natural forms of α-tocopherols are widely used in published studies. Vitamin E is a chain-breaking, lipid-soluble antioxidant present in the membrane of all cells, and immune cells contain particularly high levels of vitamin E, which protects them from oxidative damage related to high metabolic activity, as well as high PUFA content in these cells (26, 27).

#### Immunologic Effect and Mechanism

Early studies using animal models have established a clear link between vitamin E deficiency and impairment in immune functions, e.g., depressed lymphocyte proliferation in rats (28), dogs (29), lambs (30), pigs (31), and chickens (32), which can be reversed by repletion of vitamin E.

There is growing evidence to suggest that vitamin E intake meeting the current recommendation may not be optimal to the different bodily systems, or individuals at different life stages, for example, the immune system function in the elderly. Old mice fed 500 mg/kg diet (supplementation) vs. 30 mg/kg diet (adequate level as control) vitamin E for 6 wk had enhanced T cell-mediated function including delayed-type hypersensitivity (DTH) response, lymphocyte proliferation, and IL-2 production, and decreased prostaglandin (PG)E_2_ production (33). Similarly, rats fed 585 mg vs. 50 mg vitamin E/kg diet for 12 mo had higher levels of lymphocyte proliferation and IL-2 production (34). These animal study results are reproduced in several double blind, placebo controlled clinical trials. In one study, healthy individuals (≥60 y) receiving vitamin E (800 mg/d) for 1 mo showed enhancement in DTH response, T cell proliferation, and IL-2 production, and decrease in plasma lipid peroxide and PGE_2_ production (35). To examine the dose-response of vitamin E, the same group gave the elderly subjects (≥65 y) 0, 60, 200, or 800 mg/d vitamin E for 4.5 mo and found an increased DTH response from baseline in all three vitamin E groups (36). However, the 200 mg/d vitamin E group had the greatest increase compared to the placebo group, and it was also this group that had increased Ab titers to hepatitis B and tetanus vaccines (T cell-dependent Ag) from the baseline. Increased DTH response was also reported in the healthy elderly subjects (65–80 y) who had received 100 mg/d of vitamin E for 6 mo (37).

The underlying mechanisms of the immunomodulatory effects of vitamin E have been largely elucidated using animal models combined with the cell-based approaches. It is proposed that vitamin E can enhance T cell-mediated function by directly promoting membrane integrity and positively modulating the signaling events in T cells while also protecting T cell function indirectly by reducing production of T cell-suppressing factors such as PGE_2_ from macrophages as previously reviewed (38, 39). Vitamin E can reverse the age-associated reduction in activation-induced T cell expansion and IL-2 production in naïve T cells (40), and these effects are possibly mediated through its positive impact on the early events in T cell activation including formation of effective immune synapses between APC and naïve CD4^+^ T cells as well as redistribution of signaling molecules (Zap70, LAT, Vav, and PLCγ) in these immune synapses (41, 42). With regard to the indirect effects, vitamin E has been shown to inhibit PGE_2_ production. PGE_2_ suppresses T cell response by activating adenylyl cyclase, thus increasing cAMP levels (43, 44). PGE_2_ has broad effects on different components in both the innate and adaptive immune system (45–48), such as inhibiting T cell proliferation, IL-2 production, and IL-2 receptor (IL-2R) expression (46). The suppressive effect of PGE_2_ on T cells concerns inhibition of several early signaling events that occur after T cell activation (48), and for some events, the PGE_2_-induced inhibition can be prevented by vitamin E. Although how vitamin E inhibits PGE_2_ production is not completely understood, it has been shown that vitamin E can inhibit enzymatic activity of cyclooxygenases (COX) (49), which in turn might be associated with reduced production of peroxynitrite (50).

#### Clinical Relevance

Several studies have determined the protective effects of vitamin E on influenza infection in animal models. Hayek et al. (51) reported that vitamin E supplementation (500 mg/kg diet) reduced viral titers in young and old mice infected with influenza A/Port Chalmers/1/73 (H3N2) but more significantly in old mice. Similarly, Han et al. (52) reported a reduction in viral titers and symptoms after influenza infection in mice fed vitamin E, and this protective effect was associated with improved Th1 response as indicated by IFN-γ and IL-2 production. A recent study using a bacterial infection model showed that old mice fed vitamin E (500 mg/kg diet) for 4 wk had reduced pulmonary bacterial burden, lethal septicemia, and lung inflammation (neutrophil infiltration) after infection with *Streptococcus pneumoniae* (53).

Few clinical trials have directly examined the effect of vitamin E supplementation on infection in humans. In a retrospective study (54), plasma vitamin E levels in healthy people (≥60 y) were found to be negatively related to the number of past infections in these individuals; however, no correlation was present between the vitamin status and the measurements of immune function including T cell phenotype, mitogen-induced lymphocyte proliferation, and DTH. Meydani et al. reported that the healthy elderly receiving vitamin E (60, 200, or 800 mg/d for 235 d) had a non-significant (*p* < 0.09) 30% lower incidence of self-reported infections compared to those receiving the placebo (36). In a subsequent larger, double-blind, placebo-controlled trial, this group found that the elderly nursing home residents (>65 y) receiving vitamin E supplementation (200 mg/d) for 1 year had lower incidence of upper respiratory infection (RI) and common cold compared to those receiving the placebo (55). However, the controversy exists in this topic of research as studies thus far have demonstrated mixed results. In contrast to studies reviewed above, results from the Alpha-Tocopherol Beta-Carotene Cancer Prevention (ATBC) study showed positive, no effect, and even negative effect of vitamin E on pneumonia and the common cold depending on the age, smoking history, residence, and exercise, among other factors, of the subjects (56–58). The inconsistent and controversial results for vitamin E's effect on infection may be due to the confounding factors such as the difference in health conditions of participants and the intervention protocols. For instance, the ATBC study used a small dose (50 mg/d) of vitamin E vs. 200 mg/d in the study by Meydani et al. Even using the same dose, as in a double-blind trial in the Dutch elderly cohort living in the community, Graat et al. found no effect of 200 mg/d of vitamin E on the incidence of all RI, and even reported a worsening in the severity of infections (59). However, obvious differences were noted between the two studies, such as the fact that the study by Graat et al. was conducted in free living participants, and the one by Meydani et al. was conducted in managed nursing homes. It is hoped that these discrepancies may be resolved in future studies with more standardized design and better characterized populations.

### Zn

The transition metal zinc is an essential micronutrient and it is required for controlling key biological processes that affect normal growth, development, repair, metabolism, and maintenance of cell integrity and functionality (60). Its importance to immune system has been intensively studied as previously reviewed (61–63). Zinc deficiency and inadequacy are estimated to affect 30% of the world's population and contribute to 800,000 death (64). Zinc deficiency is prevalent in developing countries and it is the fifth leading risk factor for bacterial diarrhea and pneumonia (65). Inadequate intake of zinc is also present in the developed countries, in particular more common in the elderly (66, 67), which may contribute to development of immunosenescence.

#### Immunologic Effect and Mechanism

Zinc is a nutrient crucial for maintaining homeostasis of immune system. Its deficiency negatively impacts immune cell development and functions in both innate and adaptive immunity, as manifested with thymus involution and reduced number of Th1 cells, as well as impaired immune functions including lymphocyte proliferation, IL-2 production, DTH response, Ab response, natural killer (NK) cell activity, macrophage phagocytic activity, and certain functions of neutrophils [reviewed in (68–73)]. Conversely, correction of zinc deficiency by supplementation can reverse impairment in immune system (69), and reduce mortality from infectious diseases (62, 74). In addition to boosting defense-related immune functions, the importance of zinc in maintaining immune tolerance is well-recognized. Zinc has been shown to induce development of Treg cell population (75, 76), and dampen pro-inflammatory Th17 and Th9 cell differentiation (77, 78). In a related and consistent manner, zinc was shown to drive bone marrow-derived DC to develop into tolerogenic phenotype by inhibiting MHC-II expression and promoting expression of the tolerogenic programmed death-ligands (PD-L)1 and 2, tryptophan degradation, and kynurenine production leading to skewed Treg-Th17 balance in favor of Treg (79).

Although it is clear that zinc deficiency impairs immune function, proving the assumption that zinc supplementation would enhance immune response has been frustrating and full of controversy, which is more so in human studies. In animal models for zinc deficiency, zinc repletion has been shown to reverse thymic involution as indicated by an increased thymulin activity, thymus weight, absolute number of T cells in thymocytes, and thymic output in both middle-aged (12 mo) (80) and old mice (22 mo) (81, 82), as well as increase T cell mitogen PHA- or Con A-stimulated lymphocyte proliferation and NK cell activity in old mice (81). In a recent prospective clinical trial, Iovino et al. reported that multiple myeloma patients receiving a high-dose (150 mg/day) of zinc from day 5 to day 100 had significant increase of CD4^+^ naïve lymphocytes and T-cell receptor excision circle (TREC, an indicator for thymic output) (83). However, the effects of zinc supplementation on lymphocyte population are inconsistent. For example, institutionalized healthy elderly who consumed 25 mg/d zinc sulfate for 3-mo had increased numbers of activated (HLA-DR^+^) CD4^+^ and CD8^+^ T cells (84), whereas free-living elderly receiving zinc 10 mg/d zinc aspartate for 7 wk showed a reduction in activated (CD25^+^) CD4^+^ T cells (85).

Given that aging is associated with impaired immune function and increased risk of infection, and the elderly is more likely to have zinc deficiency, zinc supplementation has been identified as a part of potential solution for the immunosenescence. Thymulin is a zinc-containing thymic hormone that needs zinc to exert its biological activity (86), and serum levels of thymulin decline with aging in both mice and humans (87, 88). Similar to the results in the animal studies mentioned above (80, 81), zinc supplementation increased circulating levels of active thymulin in the elderly (66, 89, 90). Serum zinc levels were strongly correlated with the proportion of NK cells in healthy older individuals (>90 y) (91), and zinc supplementation increased NK cell cytotoxicity in both healthy elderly (90), and zinc-deficient elderly (92). Based on an *in vitro* study showing that thymulin administration improved the impaired NK cell activity in old mice, the authors suggested that thymulin may in part mediate this effect of zinc (93). Regarding the adaptive immunity, the earlier studies revealed that zinc supplementation was effective in improving DTH response (66, 94–96). More recently, zinc supplementation was shown to increase peripheral blood mononuclear cell (PBMC) mRNA expression of IL-2 and IL-2R-α (a specific subunit of IL-2R) in the elderly (97). It is suggested that zinc may influence CD4^+^ T cell polarization in favor of Th1, which involves increasing IFN-γ production through upregulation of IL-12 signaling and transcription factor T-bet activity (98). Barnett et al. recently reported that zinc supplementation (30 mg/d for 3 mo) increased serum zinc concentrations, which was correlated with the number of peripheral T cells. They also observed an increase in T cell proliferation; however this may simply reflect the larger number of T cells present in PBMC before stimulation rather than a change in capacity of T cell expansion (99).

#### Clinical Relevance

Given the importance of zinc to the immune system, in particular its boosting effect on defense-related immune responses, its impact on infection has been studied. Zinc deficiency is prevalent in children under 5 y of age in developing countries (100), and a systemic review reported that preventive zinc supplementation was associated with reduction in diarrhea and pneumonia morbidity and mortality in children (3 mo to 5 y) of developing countries (101). Guatemalan children (6–9 mo) treated with 10 mg of zinc/d as sulfate for 7 mo had decreased diarrhea by 22% but had no effect on RI incidence (102). Similarly, a large controlled trial reported that zinc supplementation (70 mg, weekly) in children (<2 y, *n* = 706) had lower incidence of pneumonia compared to the placebo group (*n* = 768) (103). After administering 75 mg of zinc/d for 3 mo to sickle-cell disease patients, who are commonly zinc deficient, the investigators found a reduction in total number of infections and upper RI, together with an increased production of IL-2 and IFN-γ in these patients (104).

Several controlled trials have investigated whether zinc supplementation is protective against infection in the elderly population. In one study supplementation with 20 mg zinc and 100 μg selenium for 2 y was associated with a significant decrease in the event of RI in institutionalized elderly (>65 y, *n* = 81) (105). Another study in an older cohort (55–87 y and 35% were zinc-deficient) supplemented with 45 mg zinc/d for 1 y showed marginally reduced incidence of common colds (*p* = 0.067) and fewer infections and fevers during the study (106). A later study by Meydani et al. showed that 29% of nursing home residents (>65 y) had low serum zinc levels (<70 μg/dL) even after receiving multi-vitamins/minerals including 7 mg zinc/d for 1 year, and compared to these individuals, those with serum zinc >70 μg/dL had lower pneumonia incidence, less total antibiotic use, and shorter duration of pneumonia and antibiotic use (107).

Since Zinc differentially affects CD4^+^ T cell populations, i.e., promoting anti-inflammatory Treg and suppressing pro-inflammatory Th17 and Th9, it is expected to mitigate autoimmune inflammatory disorders. This speculation is supported by some but not all studies. The supporting evidence includes that low serum zinc levels are associated with several prominent autoimmune diseases such as MS (108), RA (109), and T1D (110). Viewed in a larger picture, authors of a recent systematic review and meta-analysis investigated relationship between zinc status and autoimmunity using data from 62 studies that met their inclusion criteria (111). They summed up that zinc concentrations in serum (mean effect: −1.19, confidence interval: −1.26 to −1.11) and plasma (mean effect: −3.97, confidence interval: −4.08 to −3.87) of autoimmune disease patients were significantly lower compared to the controls. However, although in some cases zinc supplementation was shown to help ameliorate the disease together with relevant changes in immunological events, the causal relationship between zinc deficiency and autoimmune disease is still a matter in debate.

Inflammation is an essential response of a host to infection which helps destroy invading pathogens. However, under certain circumstance the inflammation becomes systemic so that it is harmful and even fatal to the host. A typical example of this type of systemic inflammatory response is sepsis, a syndrome characterized by organ failure resulting from over-reactive host response to infection. In human sepsis patients and in animal models, low zinc levels (probably due to internal redistribution of zinc) are associated with increased sensitivity to sepsis and fatality to infection (112), thus it is proposed that zinc supplementation might be a treatment option to improve the outcomes of sepsis. In some studies to address this issue, increasing blood zinc levels has been shown to be protective in animal sepsis models (113, 114), which is to certain degree echoed by a limited number of clinical trials, mainly in neonates (115, 116). However, no consensus is reached at present because the benefit of zinc supplementation in sepsis cannot be confirmed in other studies (62, 117). A key factor involved in this discrepancy is the fact that while immune cells on the host defense side are sensitive to the zinc status, the invading pathogens also require zinc for survival and propagation. As such, while sequestering zinc is considered a protective response to restrict pathogens, the resulting decline in serum zinc levels may compromise the immune cell functions resulting in adverse effect. The multiple physiological purposes of zinc level control in the context of infection and sepsis are a topic to be further characterized.

From the studies thus far, it is clear that children and elderly are at high risk for zinc deficiency, which is associated with the impaired immune function contributing to the increased morbidity and mortality from infections in these populations. Improving zinc status by supplementation may be helpful in addressing this problem, particularly for those with low serum zinc levels. However, given the fact that both zinc deficiency and zinc overload impair immune functions leaving a relatively narrow range for delivering benefit, plus the well-recognized heterogeneous manner in response to zinc, further studies are needed to determine the optimal zinc intake for individuals, and these studies should take into account the variations in individual genetic background as well as nutritional and health status.

### Fish Oil and n-3 PUFA

In addition to being energy-providing macronutrients, many dietary lipids, in particular PUFA, as well as their metabolic products, are capable of regulating cell functions. Of these PUFA, the marine animal-derived n-3 PUFA, composed of mainly eicosapentaenoic acid (EPA), and docosahexaenoic acid (DHA), have been intensively studied and they are known to greatly impact immune cell functions. N-6 PUFA, however, are less significant in this regard and in fact they are often used as the control for n-3 PUFA in the studies. Several recent reviews have provided comprehensive coverage for the role of n-3 PUFA in modulating both innate and adaptive immunity (118–123), thus only emerging novel research is emphasized in this review, with a focus on immunomodulatory mechanisms.

#### Immunologic Effect and Mechanism

As summarized in the above-mentioned reviews, the potent anti-inflammatory properties of n-3 PUFA is supported by their ability to inhibit production of inflammatory mediators including eicosanoids (PGE_2_, 4-series leukotrienes), pro-inflammatory cytokines (IL-1β, TNF-α, IL-6), chemokines (IL-8, MCP-1), adhesion molecules (ICAM-1, VCAM-1, selectins), platelet activating factor, and reactive oxygen and nitrogen species. In addition to inhibiting pro-inflammatory mediators, n-3 PUFA reciprocally increase the production of anti-inflammatory cytokine such as IL-10. One of the underlying mechanisms for the anti-inflammatory actions of n-3 PUFA is thought to concern modulation of gene activation. Activation of genes for most of the pro-inflammatory mediators is controlled by nuclear factor-kappa B (NF-κB), a transcription factor ubiquitous in almost all cell types. It has been demonstrated that n-3 PUFA inhibits NF-κB signaling (124, 125), possibly through interfering with the toll-like receptor 4 (TLR4) pathway and its receptor protein MyD88, activating n-3 PUFA membrane receptor GPR120, and serving as ligands to bind to and activate PPAR-γ, an anti-inflammatory transcription factor that can trans-repress NF-κB activation.

The most significant breakthrough in n-3 PUFA research is perhaps the discovery that n-3 PUFA are pro-resolution agents by serving as the precursors for several families of pre-resolving mediators, which at least include EPA-derived E-series resolvins, DHA-derived D-series resolvins, and DHA-derived protectins and maresins (126, 127). Several cell culture and animal studies have demonstrated that resolvins and protectins act to reduce neutrophil infiltration and the inflammatory response, regulate the cytokine-chemokine axis and lower the production of reactive oxygen species (127–129). Both resolvin E1 (130, 131) and maresin 1 (132) have been shown to be protective in animal models of experimental colitis, increasing survival, decreasing disease score and levels of pro-inflammatory mediators. While this suggests a potential clinical significance, there is very limited data available in humans regarding the immunomodulatory and anti-inflammatory actions of resolvins and maresins.

There is ample evidence indicating that n-3 PUFA can modulate cellular and molecular events involved in immune cell activation, particularly those related to cell-mediated immunity. Fish oil or n-3 PUFA intake has been shown to inhibit mitogen- or TCR activation-induced lymphocyte and CD4^+^ T cell proliferation, IL-2 production, and IL-2R expression, and also specific antigen-driven CD4^+^ T cell expansion under both *ex vivo* and *in vivo* conditions in animals (133–135), as well as the DTH skin response in humans (136). These T cell-inhibitory actions may be partly attributed to increased lipid peroxidation, modulation of membrane phospholipid composition, and cytoskeletal structure and disruption of lipid rafts (137–139). Changes in membrane lipid order are associated with alterations in T cell function (133, 140–142). Most recently, n-3 PUFA have been demonstrated to modulate T cell plasma membranes and oxidative phosphorylation and proliferation (139). The effect of n-3 PUFA on T cell function was also tested in fat-1 mice (137, 138), a transgenic mouse model that can endogenously synthesize n-3 PUFA, and the authors demonstrate that alteration in lipid raft formation was one potential mechanism by which n-3 PUFA suppresses T cell function. This conclusion largely concurs with the findings made in studies using dietary fish oil supplementation (133, 143).

Interestingly, the T cell-suppressive effects of n-3 PUFA are not universal to all T cells. It has been shown that n-3 PUFA inhibit Th1 and Th17 differentiation, but have little effect on Th2 and Treg development (134, 140, 144–146), or even increase Th2 and Treg populations as seen in T1D model mice (NOD mice) (147).

In addition to the direct actions on T cells, studies have suggested that n-3 PUFA may modulate the functions of APC to indirectly affect T cell functions. N-3 PUFA have been shown to inhibit APC function of spleen cells (148), monocytes/macrophages (149, 150) and dendritic cells (151–153), such as suppressing expression of MHC-II and co-stimulation molecules, activation of cognate T cells, and production of related cytokines. N-3 PUFA can also modulate B cell functions including activation, antigen presentation, cytokine production, and antibody generation (123). N-3 PUFA may target B cells to inhibit MHC-II accumulation at the immune synapse, resulting in impaired activation of cognate T cells (154, 155). N-3 PUFA appears to promote B cell activation and their production of cytokines and antibodies (156–158), which may involve Th2 cytokines, however the exact mechanism is largely elusive.

#### Clinical Relevance

Given the differential effects within the T cell population and the potent anti-inflammatory functions of n-3 PUFA, protective effects of n-3 PUFA have been reported in conditions of chronic inflammation such as asthma, IBD, including Crohn's disease and ulcerative colitis, and autoimmune disorders such as RA [reviewed in (118, 120, 159–162)].

For conditions of chronic inflammation, animal models and human studies support a beneficial role of n-3 PUFA in disease modulation. N-3 PUFA have been demonstrated to be protective in animal studies of IBD, both transgenic models (fat-1 mice) (163) and experimental models of colitis (130, 164), a chronic inflammatory condition in the gut. Yet, not all pre-clinical models support a beneficial role of n-3 PUFA on disease progression, with some animal studies indicating that large n-3 PUFA doses may exacerbate the disease (165, 166). The inconstancies in findings from animal studies, likely due to different doses of n-3 and experimental methods, need to be considered when translating conclusions to humans. In clinical trials in humans, dietary supplementation with n-3 PUFA appears to beneficially affect histological and clinical parameters of IBD (167, 168). However, a Cochrane systematic review (169) and meta-analysis (170) concluded that data was insufficient to suggest n-3 PUFA as a primary treatment for IBD suggesting that further research needs to be done regarding the efficacy of n-3 PUFA on disease progression and remission of IBD. Several randomized controlled clinical trials have demonstrated an improvement in clinical outcomes of asthma, a chronic inflammatory condition of the airways, with n-3 PUFA supplementation (171–173). Yet not all findings are consistent regarding the improvement of symptoms (174, 175), which can be related to variance in n-3 PUFA dose, population studied and study design (176). A meta-analysis and systematic review concluded that fish oil supplementation was unlikely to be beneficial in primary prevention of allergic diseases, including asthma (177), which is consistent with the conclusion of an United Sates government technical report (178).

It has also been suggested that n-3 PUFA may be clinically relevant regarding autoimmune disorders. Results from a systematic review (162) and two meta-analyses (179, 180) on marine n-3 PUFA and RA suggest that clinical outcomes related to immune function including joint swelling and pain, disease activity, and use of non-steroid anti-inflammatory drugs are consistently and modestly improved with n-3 PUFA administration. The authors of the meta-analysis suggested that EPA and DHA supplementation at a dose of >2.7 g/d for a minimum of 3 months may maximize the clinical benefits, and thus should be considered in future rials examining n-3 PUFA and RA. T1D is another organ-specific autoimmune disease involving pancreatic β cells attacked by autoreactive T cells. A retrospective study reported that long-term dietary intake of n-3 PUFA starting at 1 year of age was associated with reduced risk of developing islet autoimmunity in children with familial T1D (181). Similarly, Norwegian infants receiving cod liver oil in the first year of life was associated with a significantly lower risk of T1D, which was likely due to n-3 PUFA rather than vitamin D because no difference was observed in those receiving other vitamin D supplements (182). These results are supported by animal studies using the appropriate disease models. For example, long-term dietary intervention with n-3 PUFA in NOD (T1D model) mice reduced T1D incidence and severity, together with decreased pro-inflammatory T cell subsets (Th1, Th17) and cytokines, and increased anti-inflammatory T cell subsets (Th2, Treg) (147).

### Probiotics

Probiotics are defined as “live microorganisms that, when administered in adequate amounts, confer a health benefit on the host” (183, 184). The primary genera of probiotic microorganisms include *Lactobacillus* (*L*.), *Bifidobacterium* (*B*.), and *Streptococcus* (*S*.). *Lactobacillus* and *Bifidobacterium* have a long history of being safely used in the form of dairy products, and they are also found to be a part of the gut microbiota.

#### Immunologic Effect and Mechanism

Dietary intake of probiotics allows their intimate interaction with the gut mucosa and mucosal immune system which host the largest part of body's immune cells. Probiotics modulate immune and inflammatory response in gut through their interaction with intestinal epithelial cells (185, 186), M-cells in Peyer's patches (187, 188), and DC (189, 190). Effects of probiotics on the mucosal system are not limited to gut, with modulatory effects observed in the other locations of the mucosal system such as upper respiratory tract (191). Increasing evidence suggests that probiotics may also positively impact the systemic immune system (189, 190, 192–194). Several studies have indicated that probiotics could induce pro-inflammatory cytokines to facilitate immune response against infection, and they may also induce anti-inflammatory cytokines to mitigate the excessive inflammatory reaction leading to a balanced homeostasis [reviewed in (186, 195, 196)]. It is worth noting that the effect of probiotics on cytokine production may be strain-dependent given the mixed results showing that consuming probiotics induces IFN-α [*B. lactis* HN019, 197], reduces TNF-α [*L. rhamnosus* GG, 198] and IL-2 [*B. animalis* ssp. *Lactis* Bb12, 198], and has no effect on IFN-γ, IL-1β, and IL-2 [*L. casei*, 199].

Probiotics can benefit innate immunity by impacting intestinal epithelial cells, phagocytic APC (DC and macrophages). Epithelial cells not only serve as physical barrier but also emerge as active interphase between foreign microorganisms or food components and the body, and in doing so they participate in controlling the body's immune response (200). Some strains of probiotics can modulate mucosal immunity by colonizing on epithelium and stimulating the epithelial secretion of signaling molecules or directly acting on immune cells in the mucosal immune system, in particular DC, which protrude through epithelial junction. It is believed that probiotics play a role in maintaining homeostasis in the gut that is exposed to many foreign substances, including both harmful and harmless, by balancing the pro-inflammatory and anti-inflammatory/regulatory immune response (201). In terms of defense function, probiotic *lactobasilli* are shown to increase intestinal IgA secretion and improve the resistance to infection (202, 203). *Lactobasilli* are also shown to modulate innate immunity and DC function. Administration to mice with two *B*. strains of *lactobasilli* isolated from healthy centenarians enhanced NK cell activity and phagocytic activity of macrophages (204), and coupled with probiotics *L. fermentum* strain PL9005 and *L. plantarum* strain PL9011 enhanced the phagocytic capacity of peritoneal leukocytes (205). Mice receiving *L. paracasei* NTU 101 (10^8^ CFU/d) for 6 or 9 wk showed higher expression of DC maturation markers (MHC-II^hi^, CD80^+^, and CD86^+^) and NK group-2D (NKG2D), as well as enhanced lymphocyte proliferation in response to *L. paracasei* Ag (206), which together suggest that probiotics may enhance specific immunity by promoting APC function. Providing further support, Vidal et al. showed that following vaccination with keyhole limpet hemocyanin (KLH), old mice fed *L. paracasei* NCC2461 (1 × 10^9^ CFUs/d) for 44 d had an improved KLH-specific CD4^+^ T cell response including anti-KLH IgG2a production and DTH response (194).

Consistent with the results from animal studies, human studies have reported that certain strains of probiotics could impact the innate immunity. Healthy, older individuals receiving *B. lactis* (3 × 10^11^ CFU/d) for 6 wk had increased phagocytic and bactericidal activities of polymorphonuclear cells (PMN) in response to *Staphylococcus aureus* challenge (197), and those receiving *L. rhamnosus* HN001 (5 × 10^10^ CFU/d) or *B. lactis* HN019 (5 × 10^9^ and 5 × 10^10^ CFU/d) for 3 wk showed increased peripheral blood proportion of NK cells and their tumoricidal activity, as well as increased phagocytic activity of PBMC and PMN cells (207). The immuno-enhancing effect has been demonstrated with use of different strains of probiotics including *L. rhamnosus*, 5 × 10^10^ CFU/d (208), *L. casei* DN114001 (209), *L. lactis*, 3.4 × 10^10^ CFU/d (210), and *L. GG*, 2.6 × 10^8^ CFU/d (211).

Evidence for the beneficial effect of probiotics on adaptive immune responses largely relates to their modulatory role in promoting vigorous effector functions of both T and B cells while maintaining the regulatory functions of immune system (preventing autoimmune inflammatory response). While it is difficult to characterize how probiotics affect T cell polarization and their effector functions, including particular spectrum of cytokine production, because their effects in this regard are widely varied depending on the strains used, it appears that they promote production of Th1 cytokines (IFN-γ, IL-2, IL-12, TNF-α), Th17 cytokines (IL-17, IL-22), Treg cytokines (IL-10, TGF-β), but inhibit Th2 cytokines (IL-4) (212, 213). In animal studies, age-related decline in producing T cell cytokine IFN-α and IFN-γ by mitogen-stimulated splenocytes was reversed after administration of viable *L. bulgaricus* and *S. thermophilus* (8 × 10^8^ colony forming units (CFU)/d) for 7 d in mice (188). Similarly, administration of *B. bifidum* (5 × 10^8^ CFU/d) for 8 wk not only increased mitogen Con A-induced production of IL-2 and IFN-γ in splenocytes but also decreased systemic (serum) levels of IL-6 and TNF-α in old mice (214).

#### Clinical Relevance

Favorable effects of probiotics on both APC and cell-mediated functions suggest a potential benefit for increasing vaccination efficacy, which is particularly important in the older individuals who have lower response to vaccines than the younger individuals (215). Indeed, It has been reported that healthy nursing home residents (>70 y) have improved Ab titer against influenza vaccine and seroconversion after daily consumption of a product containing *L. casei* DN114001 (2 × 10^10^ CFU/d) and *S. thermophilus* and *L. bulgaricus* (2 × 10^10^ CFU/d) for 13 wk; however, no protective effect was found after a shorter supplementation (7 wk) in this study (216). Similarly, a short period (7 d) of *L. GG* or *L. lactis* supplementation had no effect on humoral response induced by *Salmonella typhi* oral vaccine in healthy adults (210). These results emphasize the importance of identifying optimal periods and doses of supplementation for probiotic intervention.

More relevant to clinical application, probiotics have been shown to enhance the host's resistance against infection. For example, studies have reported that fermented milk containing *Lactobacillus* reduced the duration of respiratory and gastrointestinal infections (217–219), and reduced the risk of the common cold (220). In a randomized, controlled trial in a free-living elderly cohort (*n* = 360), the participants receiving milk fermented with yogurt cultures and *L. casei* DN-114001 for 3 wk had shorter duration of winter infections (gastrointestinal and respiratory) compared to those in the control group (7 vs. 8.7 d, *n* = 180 in each group) but no difference was found in the number of illnesses (219). This beneficial effect was later confirmed in a larger trial in which healthy free-living elderly (*n* = 1,072) received milk fermented with yogurt cultures (*L. bulgaricus* & *S. thermophilus*) and *L. casei* DN114001 (2 × 10^10^ CFU/d) for 3 mo (218). Since the probiotics used in these studies contained both the strain (*L. casei* DN114001) and the yogurt cultures which include *L. bulgaricus* and *S. thermophiles*, as well as their fermented metabolites, it is difficult to distinguish the relative contributions of these components as well as the likely synergistic effects among them. There is increasing interest in investigating the effect of probiotics apart from the general effects of yogurt. Mane et al. reported that the institutionalized healthy older persons who consumed a mixture of *L. plantarum* CECT7315 and 7316 (5 × 10^8^-5 × 10^9^ CFU/d) in skim milk for 12 wk had significantly fewer incidences of infection and mortality due to pneumonia compared to those received skim milk only (221). Interestingly, this study also found that participants in the probiotic group had increased percentage of B cells, NK cells, APC, CD4^+^CD25^+^, and CD8^+^CD25^+^ phenotypes in peripheral blood cells, and most of these changes lasted 12 wk after probiotic discontinuation (221).

Beneficial effect of probiotics on the immunity and defense function has been observed in some studies but the reproducibility of this effect is still a widely recognized problem in the field. In addition, for those positive effects observed, the exact working mechanisms have not been well-elucidated. A generally accepted notion is that these effects of probiotics are related to their capability of reinforcing the intestinal barrier and helping maintain normal permeability, competing with pathogenic microorganisms in the gut for nutrients and attachment to the gut epithelium, and regulating immune cell functions to clear infection while preventing excessive response and inflammation. Probiotics exert their protective effects against infection through multiple mechanisms. A unique character separating them from other nutrients and non-nutrient phytochemicals is the fact that they are bacteria themselves, and a prominent mechanism for their anti-infection property is their direct impact on pathogens independent of immune system. They compete with pathogens for colonizing epithelium and also release antimicrobial substances together leading to an unfavorable microenvironment for pathogens.

From the experimental aspect, the *in vitro* studies can be used to assess the direct effect of probiotics on different immune cells, usually by co-culturing them and then measuring the change in phenotype and functionality of the targeted cells. In the *in vivo* setting, however, it is difficult to distinguish the direct effect from indirect effect. A main reason is that administration of particular probiotics not only changes their presence/abundance in the gut, but it is also expected to impact the gut microbiota community. Thus, study on probiotics should take into account the gut microbiota large picture. It is increasingly recognized that gut microbiota are in fact the constituents of our body and they significantly impact a variety of physiological functions including immunity.

Probiotics have also been tested in improving allergies. In a small pilot study conducted in individuals with seasonal allergic rhinitis (*n* = 10/group), Ivory et al. found that participants receiving *Lactobacillus casei* Shirota drink for 5 mo had lower antigen-induced production of IL-5, IL-6, and IFN-γ in PBMC, as well as increased IgG and decreased IgE levels in serum compared to the placebo group; however, no difference in clinical symptoms was observed (222). In a later trial with similar design but larger sample size and more comprehensive outcome measures, the same group found difference between probiotics and control groups in several immunologic parameters suggesting favorable effect of probiotics on allergy, however, they once again failed to detect difference in primary effect on clinical endpoints (223). By viewing many other trials which demonstrated mixed results, it is reasonable to conclude that evidence is lacking to support the beneficial effect of probiotics on allergy at present. As with their immuno-modulating and anti-infection effects, this may be related to several factors that should be addressed in the future as discussed in the followings.

Although promising, many claimed health benefits of probiotics have not been substantiated by intervention studies. Probiotics include a wide variety of species and they in turn are composed of many strains, either naturally occurring or intentionally modified, which have been used in different studies. It is likely that the probiotics' immune-modulating effect is strain-specific. Thus, the positive or negative findings in certain strains should not be generalized for drawing conclusions, and likewise, beneficial effects observed on certain strains cannot be extrapolated to other strains without direct experimental evidence. Additionally, the interaction among probiotics adds further challenge, which may be predicted by simply summing up their respective effects when administered individually. On the side of subjects being tested, their health status is a factor known to significantly influence the magnitude or even direction of response to a given probiotic intervention. For example, several strains of Lactobacilli and *Bifidobacteria* have been shown to differentially affect the Th1 and Th2 responses in PBMC from healthy and allergy patients (224), and *Lactobacillus* GG administration stimulated expression of phagocytosis receptors in normal healthy individuals but suppressed induction of these receptors in milk-hypersensitive individuals (211). It is also worth pointing out that results from animal studies cannot be directly extrapolated to humans before being validated by clinical trials. The other thing should in mind given the well-known fact that negative results tend to be not submitted or get rejected after submission, it is conceivable that there must be more studies than reported that have failed to prove efficacy of probiotics in favorably impacting immune function and related diseases. Nevertheless, the mechanisms underlying the reported effects of probiotics have not been well-elucidated, and obtaining such information would help identify effective probiotics for developing preventive and therapeutic strategies as well as nutritional support in targeted diseases. It is no doubt that fulfilling this task requires tremendous effort which not only involves screening individual probiotics, the combination of various strains and doses, and the timing and supplementation period needed, but also includes consideration of individual's health status and disease type.

### Green Tea and Epigallocatechin-3-Gallate (EGCG)

Green tea contains high content of catechins, accounting for 10–15% of its dry weight, which include epicatechin (EC), epicatechin-3-gallate (ECG), epigallocatechin (EGC), and epigallocatechin-3-gallate (EGCG). EGCG is the most abundant and also most biologically active, which is believed to be a primary factor responsible for green tea's health benefit. Green tea and EGCG have been shown to be effective in modulating multiple aspects of innate and adaptive immunity (225).

#### Immunologic Effect and Mechanism

In the innate immune system, *in vitro* EGCG supplementation dose-dependently reduces neutrophil migration induced by chemokine IL-8 (226), and neutrophil chemotaxis toward cytokine-induced neutrophil chemoattractant-1 (227). The oral administration of green tea extract or EGCG is shown to inhibit neutrophil recruitment to the inflammation sites in several animal studies such as mouse model of inflammatory angiogenesis (226), and rat model of ovalbumin-induced allergy (227), and to inhibit neutrophil proteolytic enzymes in a rat smoking model (228). Similarly, EGCG is also shown to inhibit monocyte migration by reducing secretion of the chemokine monocyte chemotactic protein-1 (MCP-1) and its receptor (CCR2) expression (229). Monocytes/macrophages are the primary source for most of the prominent pro-inflammatory mediators. EGCG's anti-inflammatory property is mainly drawn from its inhibitory effect on production of pro-inflammatory molecules in a variety of monocytes/macrophages cell types as previously reviewed (225). However, this is not without controversy as some investigators have reported varying results. For example, studies have shown that *in vitro* EGCG supplementation may increase production of the inflammatory mediator PGE_2_ and mRNA expression of COX-2 in RAW264.7 cells (230, 231), as well as production of IL-12p40/p70, TNF-α, and IFN-γ in murine alveolar macrophage cell line MH-S cells infected by *Legionella pneumophila* infection (232). Yet, *in vivo* supplementation showed that mice fed 1% EGCG diet produced more TNF-α, IL-6, IL-1β, and PGE_2_ in their splenocytes and macrophages as well as an elevated proportion of macrophages in spleen (233). The discrepancy in reported EGCG effect may be related to the varied experimental settings and procedural differences. Among other things, it is possible that basal levels of inflammatory status may cause a host to respond in different manner to EGCG administration and as such, the nature and magnitude of EGCG effect may vary depending on inflammation state under normal or disease condition.

DC as APC are also affected by EGCG. It has been reported that EGCG retards bone marrow-derived DC maturation and inhibits their functions as indicated by reduced ability to capture Ag (dextran), secrete IL-12, and express CD80, CD86, and MHC class I and II, culminating in impaired APC function in inducing Ag-specific T cell-mediated response (allogeneic T cell proliferation and IL-2 production) (234). Similar effects were reported in a study using human peripheral blood monocytes-derived DC (235). A very limited number of studies have examined how EGCG impacts other innate immune cells such as NK cells, mast cells, and basophils; however, they are largely cell-based studies and the results are insufficient for a meaningful speculation.

The effect of green tea/EGCG on adaptive immune functions has been relatively more intensively studied with research focusing primarily on T cell-mediated functions, especially those involving CD4^+^ T cells. Little is known regarding the humoral immunity except that *in vitro* EGCG was shown to inhibit B cell proliferation (236, 237). Wu et al. reported that *in vitro* supplementation with physiologically relevant levels of EGCG (2.5–10 μM) dose-dependently inhibits Con A-induced splenocyte proliferation, T cell division, and cell cycle progression (238). In a later study using purified T cells, the same group further showed that EGCG inhibited anti-CD3/CD28-stimulated cell division in both CD4^+^ T cells and CD8^+^ T cells but more so in the former. EGCG also inhibited antigen-specific T cell proliferation by affecting both T cells and APC while the direct effect on T cells appeared to be predominant (239). The T cell-suppressive effect of EGCG was confirmed in the *in vivo* study in which mice were fed a diet containing 0.3% EGCG for 6 wk (239). *In vitro* EGCG supplementation has been shown to decrease IL-2 production in response to allogeneic stimulator cells (240), production of IL-2, TNF-α, and IFN-γ in *Staphylococcus* enterotoxin B-stimulated human PBMC (241), and IFN-γ production in Con A-stimulated mouse splenocytes (238), or anti-CD3/CD28-stimulated mouse CD4^+^ T cells (242). However, some other studies reported different results which include EGCG-induced upregulation in mRNA levels of Th1 cytokines (IL-2 and IFN-γ) and Th2 cytokines (IL-5 and IL-13) in Jurkat cells (243), and increased IL-2 production in response to PMA and PHA in human PBMC (244). These discrepant findings may be related to the different experimental conditions such as cell type, EGCG concentration, and stimulation condition used. In addition, sometimes altered cytokine levels may not necessarily tell the situation in their synthesis. For example, EGCG did not affect IL-2 levels in the culture of T cells stimulated for 24 h or shorter, but caused a dose-dependent elevation of IL-2 in 48 h cultures (239). Further tests showed that EGCG did not affect IL-2 synthesis as confirmed by intracellular staining and mRNA levels, but instead, it reduced IL-2R expression, which together suggest that higher levels of IL-2 might result from increased IL-2 accumulation due to a reduction in IL-2R-mediated IL-2 internalization and utilization (239). This hypothesis was supported by a later study showing that EGCG-mediated inhibition of IL-2R involves all three IL-2R subunits: IL-2Rα, IL-2Rβ (CD122, shared with IL-15R), and γc (CD132, shared with IL-7R and IL-15R), as well as their downstream signaling events (245).

The mechanisms for EGCG-induced inhibition of cytokine production and T cell proliferation are yet to be clearly elucidated; however, some evidence from *in vitro* studies suggests an involvement of EGCG-induced interference with early signaling events in T cell activation. It has been reported that in Jurkat T cells, EGCG inhibits the early stages of the T cell signaling pathways including activation of Zap70, LAT, phospholipase Cγ1, ERK, MAPK, and transcription factor AP-1 (246); the cyclin dependent kinase inhibitor p27^Kip1^, a negative regulator of cell cycle progression, was identified as a molecular target of EGCG (247).

As mentioned above, EGCG has a strong potency in inhibiting CD4^+^ T cell proliferation and appears to alter T cell differentiation. Recent studies have revealed that EGCG differentially impacts development of CD4^+^ T cell subpopulations. By incubating naïve CD4^+^ T cells under different Th differentiation conditions in the presence of 10 μM EGCG, Wang et al. found that EGCG suppressed CD4^+^ T cells polarization toward Th1 and Th17 subsets, and also partly prevented IL-6-induced suppression of Treg development, but had no effect on Th2 differentiation (242).

#### Clinical Relevance

From the reported effects of EGCG on immune cell functions, particularly its anti-inflammatory, T cell-suppressing, and differentiation-modulating effects on T cell subset development, EGCG appears to have a potential benefit in clinical application for preventing and mitigating T cell-mediated autoimmune diseases. Indeed, administration of EGCG has been shown to improve several autoimmune diseases in respective rodent models including experimental autoimmune encephalomyelitis (EAE, for human multiple sclerosis, or MS), collagen- or Ag-induced arthritis (for RA), the chemically-induced colitis (for IBD), and the non-obese diabetic mouse strains (for Sjogren's syndrome) [reviewed in (225, 248)]. In the earlier studies, the beneficial effect of EGCG in these autoimmune diseases is largely attributed to EGCG's anti-inflammatory properties. Promoted by the development of research on CD4^+^ T cell subpopulations as well as the evolving theory for their involvement in autoimmune pathogenesis, the more recent studies have generated new evidence to suggest that desirable effect of EGCG on autoantigen-induced T cell activation, differentiation, and effector functions during the initiation and development of autoimmunity may represent an important mechanism underlying the EGCG's beneficial effect in autoimmune disease. However, thus far almost all the evidence is from animal studies, and the efficacy and safety for EGCG's clinical application in human diseases remain to be established.

## Conclusions

**Figure 1:**
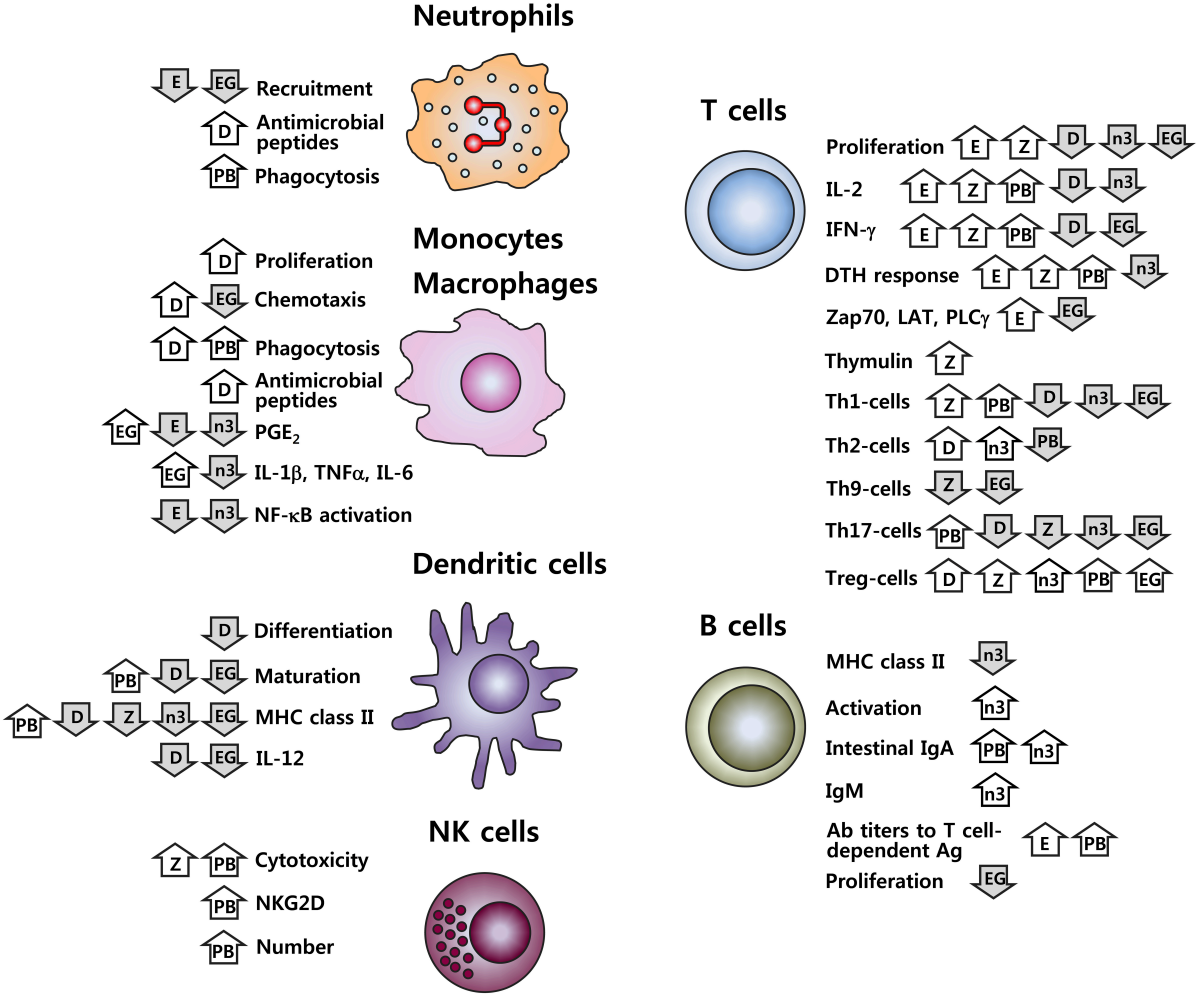
Immune cell functions affected by vitamins D and E, zinc, n-3 PUFA, probiotics, and EGCG. D, vitamin D; E, vitamin E; Z, zinc; n-3, n-3 PUFA; PB, probiotics; EG, EGCG; 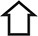, increase; 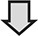, decrease. Effects of probiotics cited here are for some strains; given the strain-specific nature for the effects of probiotics, these results should not be generalized.

It is well-established that nutritional inadequacy greatly impairs the functioning of the immune system. In addition, it is increasingly recognized that nutrient intake, above what is currently recommended, may beneficially affect immune function, modulate chronic inflammatory and autoimmune conditions, and decrease infection risk. This includes both macronutrients (lipids such as n-3 PUFA) and micronutrients (zinc, vitamin D and vitamin E), in addition to phytochemicals and functional foods (probiotics and green tea). Many of these nutritive and non-nutritive food components are related in their functions to maintain or improve immune function including inhibition of pro-inflammatory mediators, promotion of anti-inflammatory functions, modulation of cell-mediated immunity, alteration of APC function, and communication between the innate and adaptive immune systems. Figure 1 provides a schematic summary of the immuno-modulating features for the six types of food components discussed in this review. It should be in mind that this simplified picture cannot cover complete outcomes in the respective research, nor can it accurately reflect the controversial issues present. It is particularly worth mentioning that effects of probiotics cited in the figure are based on the results for some strains. Considering the well-recognized strain-specific feature of the biological effects of probiotics, caution should be taken in data interpretation and extrapolation.

The properties of the nutrients, phytochemicals, and functional foods in modulating immune function have significant implications for inflammation-mediated conditions. Both animal and human studies have presented promising findings suggesting a clinical benefit of vitamin D, n-3 PUFA and EGCG in chronic inflammatory conditions, n-3 PUFA and EGCG in autoimmune disorders, and vitamin D, vitamin E, zinc and probiotics in protection against infection. However, the discrepancy in results from many studies adds the challenge and complexity of nutritional immunology research; as the result, there is no clear consensus at this time regarding the clinical relevance of these dietary components. In some cases, results in human studies are not always consistent with pre-clinical animal models, or the immunomodulatory effects have not yet been examined in humans. Moreover, there is great variation among human study designs, the doses used, and the populations of study, demonstrating a need for more standardized clinical trial designs, better characterized populations, more information for determining the intervention dose used, and more meaningful outcome measurements chosen. Particularly for zinc, vitamin E, n-3 PUFA and probiotics, clearly there is need to establish the optimal doses for maximum clinical benefits, which may likely differ depending on the age, genetic background, and nutritional and health status of the population of study.

## Author Contributions

All authors listed have made a substantial, direct and intellectual contribution to the work, and approved it for publication.

### Conflict of Interest Statement

The authors declare that the research was conducted in the absence of any commercial or financial relationships that could be construed as a potential conflict of interest.

## References

[1] Baeke F Takiishi T Korf H Gysemans C Mathieu C. Vitamin D: modulator of the immune system. Curr Opin Pharmacol. (2010) 10:482–96. 10.1016/j.coph.2010.04.001 20427238

[2] Gombart AF. The vitamin D-antimicrobial peptide pathway and its role in protection against infection. Future Microbiol. (2009) 4:1151–65. 10.2217/fmb.09.87 19895218 PMC2821804

[3] Ohta M Okabe T Ozawa K Urabe A Takaku F. 1 alpha,25-Dihydroxyvitamin D3 (calcitriol) stimulates proliferation of human circulating monocytes *in vitro*. FEBS Lett. (1985) 185:9–13. 10.1016/0014-5793(85)80730-4 3838944

[4] Xu H Soruri A Gieseler RK Peters JH. 1,25-Dihydroxyvitamin D3 exerts opposing effects to IL-4 on MHC class-II antigen expression, accessory activity, and phagocytosis of human monocytes. Scand J Immunol. (1993) 38:535–40. 10.1111/j.1365-3083.1993.tb03237.x 8256111

[5] Gombart AF Borregaard N Koeffler HP. Human cathelicidin antimicrobial peptide (CAMP) gene is a direct target of the vitamin D receptor and is strongly up-regulated in myeloid cells by 1,25-dihydroxyvitamin D3. FASEB J. (2005) 19:1067–77. 10.1096/fj.04-3284com 15985530

[6] Liu PT Stenger S Li H Wenzel L Tan BH Krutzik SR. Toll-like receptor triggering of a vitamin D-mediated human antimicrobial response. Science (2006) 311:1770–3. 10.1126/science.1123933 16497887

[7] Wang TT Nestel FP Bourdeau V Nagai Y Wang Q Liao J. Cutting edge: 1,25-dihydroxyvitamin D3 is a direct inducer of antimicrobial peptide gene expression. J Immunol. (2004) 173:2909–12. 10.4049/jimmunol.173.5.2909 15322146

[8] Provvedini DM Tsoukas CD Deftos LJ Manolagas SC. 1,25-dihydroxyvitamin D3 receptors in human leukocytes. Science (1983) 221:1181–3. 10.1126/science.6310748 6310748

[9] Lemire JM Adams JS Sakai R Jordan SC. 1 alpha,25-dihydroxyvitamin D3 suppresses proliferation and immunoglobulin production by normal human peripheral blood mononuclear cells. J Clin Invest. (1984) 74:657–61. 10.1172/JCI111465 6611355 PMC370520

[10] Rigby WF Stacy T Fanger MW. Inhibition of T lymphocyte mitogenesis by 1,25-dihydroxyvitamin D3 (calcitriol). J Clin Invest. (1984) 74:1451–5. 10.1172/JCI111557 6332829 PMC425314

[11] Kongsbak M Levring TB Geisler C von Essen MR. The vitamin d receptor and T cell function. Front Immunol. (2013) 4:148. 10.3389/fimmu.2013.00148 23785369 PMC3684798

[12] Mora JR Iwata M von Andrian UH. Vitamin effects on the immune system: vitamins A and D take centre stage. Nat Rev Immunol. (2008) 8:685–98. 10.1038/nri2378 19172691 PMC2906676

[13] Rigby WF Denome S Fanger MW. Regulation of lymphokine production and human T lymphocyte activation by 1,25-dihydroxyvitamin D3. Specific inhibition at the level of messenger RNA. J Clin Invest. (1987) 79:1659–64. 10.1172/JCI113004 2884234 PMC424495

[14] Alroy I Towers TL Freedman LP. Transcriptional repression of the interleukin-2 gene by vitamin D3: direct inhibition of NFATp/AP-1 complex formation by a nuclear hormone receptor. Mol Cell Biol. (1995) 15:5789–99. 10.1128/MCB.15.10.5789 7565732 PMC230831

[15] Cippitelli M Santoni A. Vitamin D3: a transcriptional modulator of the interferon-gamma gene. Eur J Immunol. (1998) 28:3017–30. 10.1002/(SICI)1521-4141(199810)28:10<3017::AID-IMMU3017>;3.0.CO;2-6 9808170

[16] Wei R Christakos S. Mechanisms underlying the regulation of innate and adaptive immunity by Vitamin D. Nutrients (2015) 7:8251–60. 10.3390/nu7105392 26404359 PMC4632412

[17] Bscheider M Butcher EC. Vitamin D immunoregulation through dendritic cells. Immunology (2016) 148:227–36. 10.1111/imm.12610 27040466 PMC4913286

[18] Penna G Adorini L. 1 Alpha,25-dihydroxyvitamin D3 inhibits differentiation, maturation, activation, and survival of dendritic cells leading to impaired alloreactive T cell activation. J Immunol. (2000) 164:2405–11. 10.4049/jimmunol.164.5.2405 10679076

[19] Kearns MD Alvarez JA Seidel N Tangpricha V. Impact of vitamin D on infectious disease. Am J Med Sci. (2015) 349:245–62. 10.1097/MAJ.0000000000000360 25334038 PMC4346469

[20] Kroner Jde C Sommer A Fabri M Vitamin D every day to keep the infection away? Nutrients (2015) 7:4170–88. 10.3390/nu7064170 26035244 PMC4488779

[21] Zittermann A Pilz S Hoffmann H Marz W. Vitamin D and airway infections: a European perspective. Eur J Med Res. (2016) 21:14. 10.1186/s40001-016-0208-y 27009076 PMC4806418

[22] Colotta F Jansson B Bonelli F. Modulation of inflammatory and immune responses by vitamin D. J Autoimmun. (2017) 85:78–97. 10.1016/j.jaut.2017.07.007 28733125

[23] Dankers W Colin EM van Hamburg JP Lubberts E. Vitamin D in autoimmunity: molecular mechanisms and therapeutic potential. Front Immunol. (2016) 7:697. 10.3389/fimmu.2016.00697 28163705 PMC5247472

[24] Agmon-Levin N Theodor E Segal RM Shoenfeld Y. Vitamin D in systemic and organ-specific autoimmune diseases. Clin Rev Allergy Immunol. (2013) 45:256–66. 10.1007/s12016-012-8342-y 23238772

[25] Antico A Tampoia M Tozzoli R Bizzaro N. Can supplementation with vitamin D reduce the risk or modify the course of autoimmune diseases? A systematic review of the literature. Autoimmun Rev. (2012) 12:127–36. 10.1016/j.autrev.2012.07.007 22776787

[26] Coquette A Vray B Vanderpas J. Role of vitamin E in the protection of the resident macrophage membrane against oxidative damage. Arch Int Physiol Biochim. (1986) 94:S29–34. 2440399

[27] Hatam LJ Kayden HJ. A high-performance liquid chromatographic method for the determination of tocopherol in plasma and cellular elements of the blood. J Lipid Res. (1979) 20:639–45. 490041

[28] Eskew ML Scholz RW Reddy CC Todhunter DA Zarkower A. Effects of vitamin E and selenium deficiencies on rat immune function. Immunology (1985) 54:173–80. 3972431 PMC1454841

[29] Langweiler M Schultz RD Sheffy BE. Effect of vitamin E deficiency on the proliferative response of canine lymphocytes. Am J Vet Res. (1981) 42:1681–5. 7325428

[30] Turner RJ Finch JM. Immunological malfunctions associated with low selenium-vitamin E diets in lambs. J Comp Pathol. (1990) 102:99–109. 10.1016/S0021-9975(08)80012-6 2312800

[31] Jensen M Fossum C Ederoth M Hakkarainen RV. The effect of vitamin E on the cell-mediated immune response in pigs. Zentralbl Veterinarmed B (1988) 35:549–55. 10.1111/j.1439-0450.1988.tb00528.x 3188729

[32] Chang WP Hom JS Dietert RR Combs GF Jr Marsh JA. Effect of dietary vitamin E and selenium deficiency on chicken splenocyte proliferation and cell surface marker expression. Immunopharmacol Immunotoxicol. (1994) 16:203–23. 10.3109/08923979409007091 8077607

[33] Meydani SN Meydani M Verdon CP Shapiro AA Blumberg JB Hayes KC. Vitamin E supplementation suppresses prostaglandin E1(2) synthesis and enhances the immune response of aged mice. Mech Ageing Dev. (1986) 34:191–201. 10.1016/0047-6374(86)90034-5 3487685

[34] Sakai S Moriguchi S. Long-term feeding of high vitamin E diet improves the decreased mitogen response of rat splenic lymphocytes with aging. J Nutr Sci Vitaminol. (1997) 43:113–22. 10.3177/jnsv.43.113 9151245

[35] Meydani SN Barklund MP Liu S Meydani M Miller RA Cannon JG. Vitamin E supplementation enhances cell-mediated immunity in healthy elderly subjects. Am J Clin Nutr. (1990) 52:557–63. 10.1093/ajcn/52.3.557 2203257

[36] Meydani SN Meydani M Blumberg JB Leka LS Siber G Loszewski R. Vitamin E supplementation and *in vivo* immune response in healthy elderly subjects. A randomized controlled trial. JAMA (1997) 277:1380–6. 10.1001/jama.1997.03540410058031 9134944

[37] Pallast EG Schouten EG de Waart FG Fonk HC Doekes G von Blomberg BM. Effect of 50- and 100-mg vitamin E supplements on cellular immune function in noninstitutionalized elderly persons. Am J Clin Nutr. (1999) 69:1273–81. 10.1093/ajcn/69.6.1273 10357750

[38] Meydani SN Han SN Wu D. Vitamin E and immune response in the aged: molecular mechanisms and clinical implications. Immunol Rev. (2005) 205:269–84. 10.1111/j.0105-2896.2005.00274.x 15882360 PMC7158858

[39] Wu D Meydani SN. Age-associated changes in immune and inflammatory responses: impact of vitamin E intervention. J Leukoc Biol. (2008) 84:900–14. 10.1189/jlb.0108023 18596135 PMC2538592

[40] Adolfsson O Huber BT Meydani SN Vitamin E-enhanced IL-2 production in old mice: naive but not memory T cells show increased cell division cycling and IL-2-producing capacity. J Immunol. (2001) 167:3809–17. 10.4049/jimmunol.167.7.3809 11564798

[41] Marko MG Ahmed T Bunnell SC Wu D Chung H Huber BT. Age-associated decline in effective immune synapse formation of CD4(+) T cells is reversed by vitamin E supplementation. J Immunol. (2007) 178:1443–9. 10.4049/jimmunol.178.3.1443 17237392

[42] Marko MG Pang HJ Ren Z Azzi A Huber BT Bunnell SC. Vitamin E reverses impaired linker for activation of T cells activation in T cells from aged C57BL/6 mice. J Nutr. (2009) 139:1192–7. 10.3945/jn.108.103416 19403707 PMC2714384

[43] Smith JW Steiner AL Newberry WM Jr Parker CW. Cyclic adenosine 3′,5′-monophosphate in human lymphocytes. Alterations after phytohemagglutinin stimulation. J Clin Invest. (1971) 50:432–41. 10.1172/JCI106510 4395563 PMC291939

[44] Smith JW Steiner AL Parker CW. Human lymphocytic metabolism. Effects of cyclic and noncyclic nucleotides on stimulation by phytohemagglutinin. J Clin Invest. (1971) 50:442–8. 10.1172/JCI106511 4322078 PMC291940

[45] Harris SG Padilla J Koumas L Ray D Phipps RP. Prostaglandins as modulators of immunity. Trends Immunol. (2002) 23:144–50. 10.1016/S1471-4906(01)02154-8 11864843

[46] Kalinski P. Regulation of immune responses by prostaglandin E2. J Immunol. (2012) 188:21–8. 10.4049/jimmunol.1101029 22187483 PMC3249979

[47] Rocca B FitzGerald GA. Cyclooxygenases and prostaglandins: shaping up the immune response. Int Immunopharmacol. (2002) 2:603–30. 10.1016/S1567-5769(01)00204-1 12013502

[48] Sreeramkumar V Fresno M Cuesta N Prostaglandin E2 and T cells: friends or foes? Immunol Cell Biol. (2012) 90:579–86. 10.1038/icb.2011.75 21946663 PMC3389798

[49] Wu D Mura C Beharka AA Han SN Paulson KE Hwang D. Age-associated increase in PGE2 synthesis and COX activity in murine macrophages is reversed by vitamin E. Am J Physiol. (1998) 275:C661–8. 10.1152/ajpcell.1998.275.3.C661 9730949

[50] Beharka AA Wu D Serafini M Meydani SN. Mechanism of vitamin E inhibition of cyclooxygenase activity in macrophages from old mice: role of peroxynitrite. Free Radic Biol Med. (2002) 32:503–11. 10.1016/S0891-5849(01)00817-6 11958951

[51] Hayek MG Taylor SF Bender BS Han SN Meydani M Smith DE. Vitamin E supplementation decreases lung virus titers in mice infected with influenza. J Infect Dis. (1997) 176:273–6. 10.1086/517265 9207381

[52] Han SN Wu D Ha WK Beharka A Smith DE Bender BS. Vitamin E supplementation increases T helper 1 cytokine production in old mice infected with influenza virus. Immunology (2000) 100:487–93. 10.1046/j.1365-2567.2000.00070.x 10929076 PMC2327029

[53] Bou Ghanem EN Clark S Du X Wu D Camilli A Leong JM The alpha-tocopherol form of vitamin E reverses age-associated susceptibility to *Streptococcus pneumoniae* Lung infection by modulating pulmonary neutrophil recruitment. J Immunol. (2015) 194:1090–9. 10.4049/jimmunol.1402401 25512603 PMC4834212

[54] Chavance M Herbeth B Fournier C Janot C Vernhes G. Vitamin status, immunity and infections in an elderly population. Eur J Clin Nutr. (1989) 43:827–35. 2627929

[55] Meydani SN Leka LS Fine BC Dallal GE Keusch GT Singh MF. Vitamin E and respiratory tract infections in elderly nursing home residents: a randomized controlled trial. JAMA (2004) 292:828–36. 10.1001/jama.292.7.828 15315997 PMC2377357

[56] Hemila H Kaprio J. Subgroup analysis of large trials can guide further research: a case study of vitamin E and pneumonia. Clin Epidemiol. (2011) 3:51–9. 10.2147/CLEP.S16114 21386974 PMC3046185

[57] Hemila H Virtamo J Albanes D Kaprio J. Vitamin E and beta-carotene supplementation and hospital-treated pneumonia incidence in male smokers. Chest (2004) 125:557–65. 10.1378/chest.125.2.557 14769738

[58] Hemila H Virtamo J Albanes D Kaprio J. The effect of vitamin E on common cold incidence is modified by age, smoking and residential neighborhood. J Am Coll Nutr. (2006) 25:332–9. 10.1080/07315724.2006.10719543 16943455

[59] Graat JM Schouten EG Kok FJ. Effect of daily vitamin E and multivitamin-mineral supplementation on acute respiratory tract infections in elderly persons: a randomized controlled trial. JAMA (2002) 288:715–21. 10.1001/jama.288.6.715 12169075

[60] Prasad AS. Discovery of human zinc deficiency: its impact on human health and disease. Adv Nutr. (2013) 4:176–90. 10.3945/an.112.003210 23493534 PMC3649098

[61] Wessels I Maywald M Rink L. Zinc as a gatekeeper of immune function. Nutrients (2017) 9:E1286. 10.3390/nu9121286 29186856 PMC5748737

[62] Gammoh NZ Rink L. Zinc in infection and inflammation. Nutrients (2017) 9:E624. 10.3390/nu9060624 28629136 PMC5490603

[63] Maywald M Wessels I Rink L. Zinc signals and immunity. Int J Mol Sci (2017) 18:E2222. 10.3390/ijms18102222 29064429 PMC5666901

[64] Caulfield LE Black RE. Zinc Deficiency (2004). WHO: Geneva, p. 257–80.

[65] Wessells KR Brown KH. Estimating the global prevalence of zinc deficiency: results based on zinc availability in national food supplies and the prevalence of stunting. PLoS ONE (2012) 7:e50568. 10.1371/journal.pone.0050568 23209782 PMC3510072

[66] Prasad AS Fitzgerald JT Hess JW Kaplan J Pelen F Dardenne M. Zinc deficiency in elderly patients. Nutrition (1993) 9:218–24. 8353362

[67] Sandstead HH Henriksen LK Greger JL Prasad AS Good RA. Zinc nutriture in the elderly in relation to taste acuity, immune response, and wound healing. Am J Clin Nutr. (1982) 36:1046–59. 10.1093/ajcn/36.5.1046 6765070

[68] Mocchegiani E Malavolta M. NK and NKT cell functions in immunosenescence. Aging Cell (2004) 3:177–84. 10.1111/j.1474-9728.2004.00107.x 15268751

[69] Haase H Rink L. The immune system and the impact of zinc during aging. Immun Ageing (2009) 6:9. 10.1186/1742-4933-6-9 19523191 PMC2702361

[70] Keen CL Gershwin ME. Zinc deficiency and immune function. Annu Rev Nutr. (1990) 10:415–31. 10.1146/annurev.nu.10.070190.002215 2200472

[71] Mitchell WA Meng I Nicholson SA Aspinall R. Thymic output, ageing and zinc. Biogerontology (2006) 7:461–70. 10.1007/s10522-006-9061-7 16964524

[72] Prasad AS. Effects of zinc deficiency on Th1 and Th2 cytokine shifts. J Infect Dis. (2000) 182(Suppl. 1):S62–8. 10.1086/315916 10944485

[73] Prasad AS. Zinc in human health: effect of zinc on immune cells. Mol Med. (2008) 14:353–7. 10.2119/2008-00033.Prasad 18385818 PMC2277319

[74] Fischer Walker C Black RE Zinc and the risk for infectious disease. Annu Rev Nutr. (2004) 24:255–75. 10.1146/annurev.nutr.23.011702.073054 15189121

[75] Rosenkranz E Maywald M Hilgers RD Brieger A Clarner T Kipp M. Induction of regulatory T cells in Th1-/Th17-driven experimental autoimmune encephalomyelitis by zinc administration. J Nutr Biochem. (2016) 29:116–23. 10.1016/j.jnutbio.2015.11.010 26895672

[76] Rosenkranz E Metz CH Maywald M Hilgers RD Wessels I Senff T. Zinc supplementation induces regulatory T cells by inhibition of Sirt-1 deacetylase in mixed lymphocyte cultures. Mol Nutr Food Res. (2016) 60:661–71. 10.1002/mnfr.201500524 26614004

[77] Kitabayashi C Fukada T Kanamoto M Ohashi W Hojyo S Atsumi T. Zinc suppresses Th17 development via inhibition of STAT3 activation. Int Immunol. (2010) 22:375–86. 10.1093/intimm/dxq017 20215335

[78] Maywald M Wang F Rink L. Zinc supplementation plays a crucial role in T helper 9 differentiation in allogeneic immune reactions and non-activated T cells. J Trace Elem Med Biol. (2018) 50:482–8. 10.1016/j.jtemb.2018.02.004 29439842

[79] George MM Subramanian Vignesh K Landero Figueroa JA Caruso JA Deepe GS Jr. Zinc induces dendritic cell tolerogenic phenotype and skews regulatory T Cell-Th17 balance. J Immunol. (2016) 197:1864–76. 10.4049/jimmunol.1600410 27465530 PMC4992588

[80] Dardenne M Boukaiba N Gagnerault MC Homo-Delarche F Chappuis P Lemonnier D. Restoration of the thymus in aging mice by *in vivo* zinc supplementation. Clin Immunol Immunopathol. (1993) 66:127–35. 10.1006/clin.1993.1016 8453784

[81] Mocchegiani E Santarelli L Muzzioli M Fabris N. Reversibility of the thymic involution and of age-related peripheral immune dysfunctions by zinc supplementation in old mice. Int J Immunopharmacol. (1995) 17:703–18. 10.1016/0192-0561(95)00059-B 8582782

[82] Wong CP Song Y Elias VD Magnusson KR Ho E. Zinc supplementation increases zinc status and thymopoiesis in aged mice. J Nutr. (2009) 139:1393–7. 10.3945/jn.109.106021 19474155 PMC2696991

[83] Iovino L Mazziotta F Carulli G Guerrini F Morganti R Mazzotti V. High-dose zinc oral supplementation after stem cell transplantation causes an increase of TRECs and CD4+ naive lymphocytes and prevents TTV reactivation. Leuk Res. (2018) 70:20–24. 10.1016/j.leukres.2018.04.016 29747074

[84] Fortes C Forastiere F Agabiti N Fano V Pacifici R Virgili F. The effect of zinc and vitamin A supplementation on immune response in an older population. J Am Geriatr Soc. (1998) 46:19–26. 10.1111/j.1532-5415.1998.tb01008.x 9434661

[85] Kahmann L Uciechowski P Warmuth S Malavolta M Mocchegiani E Rink L. Effect of improved zinc status on T helper cell activation and TH1/TH2 ratio in healthy elderly individuals. Biogerontology (2006) 7:429–35. 10.1007/s10522-006-9058-2 16967204

[86] Dardenne M Pleau JM Nabarra B Lefrancier P Derrien M Choay J. Contribution of zinc and other metals to the biological activity of the serum thymic factor. Proc Natl Acad Sci USA. (1982) 79:5370–3. 10.1073/pnas.79.17.5370 6957870 PMC346898

[87] Bach JF Papiernik M Levasseur P Dardenne M Barois A Le Brigand H. Evidence for a serum-factor secreted by the human thymus. Lancet (1972) 2:1056–8. 10.1016/S0140-6736(72)92339-2 4117378

[88] Iwata T Incefy GS Tanaka T Fernandes G Menendez-Botet CJ Pih K. Circulating thymic hormone levels in zinc deficiency. Cell Immunol. (1979) 47:100–5. 10.1016/0008-8749(79)90318-6 509529

[89] Boukaiba N Flament C Acher S Chappuis P Piau A Fusselier M. A physiological amount of zinc supplementation: effects on nutritional, lipid, and thymic status in an elderly population. Am J Clin Nutr. (1993) 57:566–72. 10.1093/ajcn/57.4.566 8460613

[90] Mocchegiani E Muzzioli M Giacconi R Cipriano C Gasparini N Franceschi C. Metallothioneins/PARP-1/IL-6 interplay on natural killer cell activity in elderly: parallelism with nonagenarians and old infected humans. Effect of zinc supply. Mech Ageing Dev. (2003) 124:459–68. 10.1016/S0047-6374(03)00023-X 12714254

[91] Ravaglia G Forti P Maioli F Bastagli L Facchini A Mariani E. Effect of micronutrient status on natural killer cell immune function in healthy free-living subjects aged >/ = 90 y. Am J Clin Nutr. (2000) 71:590–8. 10.1093/ajcn/71.2.590 10648276

[92] Mocchegiani E Giacconi R Costarelli L Muti E Cipriano C Tesei S. Zinc deficiency and IL-6−174G/C polymorphism in old people from different European countries: effect of zinc supplementation. ZINCAGE study. Exp Gerontol. (2008) 43:433–44. 10.1016/j.exger.2008.01.001 18267353

[93] Muzzioli M Mocchegiani E Bressani N Bevilacqua P Fabris N. *In vitro* restoration by thymulin of NK activity of cells from old mice. Int J Immunopharmacol. (1992) 14:57–61. 10.1016/0192-0561(92)90105-T 1582734

[94] Cossack ZT. T-lymphocyte dysfunction in the elderly associated with zinc deficiency and subnormal nucleoside phosphorylase activity: effect of zinc supplementation. Eur J Cancer Clin Oncol. (1989) 25:973–6. 10.1016/0277-5379(89)90156-9 2502416

[95] Duchateau J Delepesse G Vrijens R Collet H. Beneficial effects of oral zinc supplementation on the immune response of old people. Am J Med. (1981) 70:1001–4. 10.1016/0002-9343(81)90849-4 6972165

[96] Wagner PA Jernigan JA Bailey LB Nickens C Brazzi GA. Zinc nutriture and cell-mediated immunity in the aged. Int J Vitam Nutr Res. (1983) 53:94–101. 6853062

[97] Prasad AS Bao B Beck FW Sarkar FH. Correction of interleukin-2 gene expression by in vitro zinc addition to mononuclear cells from zinc-deficient human subjects: a specific test for zinc deficiency in humans. Transl Res. (2006) 148:325–33. 10.1016/j.trsl.2006.07.008 17162254

[98] Bao B Prasad AS Beck FW Bao GW Singh T Ali S. Intracellular free zinc up-regulates IFN-gamma and T-bet essential for Th1 differentiation in Con-A stimulated HUT-78 cells. Biochem Biophys Res Commun. (2011) 407:703–7. 10.1016/j.bbrc.2011.03.084 21439265 PMC3142693

[99] Barnett JB Dao MC Hamer DH Kandel R Brandeis G Wu D. Effect of zinc supplementation on serum zinc concentration and T cell proliferation in nursing home elderly: a randomized, double-blind, placebo-controlled trial. Am J Clin Nutr. (2016) 103:942–51. 10.3945/ajcn.115.115188 26817502

[100] Gibson RS Ferguson EL. Assessment of dietary zinc in a population. Am J Clin Nutr. (1998) 68:430S−4S. 10.1093/ajcn/68.2.430S 9701157

[101] Yakoob MY Theodoratou E Jabeen A Imdad A Eisele TP Ferguson J. Preventive zinc supplementation in developing countries: impact on mortality and morbidity due to diarrhea, pneumonia and malaria. BMC Public Health (2011) 11(Suppl. 3):S23. 10.1186/1471-2458-11-S3-S23 21501441 PMC3231897

[102] Ruel MT Rivera JA Santizo MC Lonnerdal B Brown KH. Impact of zinc supplementation on morbidity from diarrhea and respiratory infections among rural Guatemalan children. Pediatrics (1997) 99:808–13. 10.1542/peds.99.6.808 9164774

[103] Brooks WA Santosham M Naheed A Goswami D Wahed MA Diener-West M. Effect of weekly zinc supplements on incidence of pneumonia and diarrhoea in children younger than 2 years in an urban, low-income population in Bangladesh: randomised controlled trial. Lancet (2005) 366:999–1004. 10.1016/S0140-6736(05)67109-7 16168782

[104] Bao B Prasad AS Beck FW Snell D Suneja A Sarkar FH. Zinc supplementation decreases oxidative stress, incidence of infection, and generation of inflammatory cytokines in sickle cell disease patients. Transl Res. (2008) 152:67–80. 10.1016/j.trsl.2008.06.001 18674741

[105] Girodon F Lombard M Galan P Brunet-Lecomte P Monget AL Arnaud J. Effect of micronutrient supplementation on infection in institutionalized elderly subjects: a controlled trial. Ann Nutr Metab. (1997) 41:98–107. 10.1159/000177984 9267584

[106] Prasad AS Beck FW Bao B Fitzgerald JT Snell DC Steinberg JD. Zinc supplementation decreases incidence of infections in the elderly: effect of zinc on generation of cytokines and oxidative stress. Am J Clin Nutr. (2007) 85:837–44. 10.1093/ajcn/85.3.837 17344507

[107] Meydani SN Barnett JB Dallal GE Fine BC Jacques PF Leka LS. Serum zinc and pneumonia in nursing home elderly. Am J Clin Nutr. (2007) 86:1167–73. 10.1093/ajcn/86.4.1167 17921398 PMC2323679

[108] Williams CM Lines CM McKay EC. Iron and zinc status in multiple sclerosis patients with pressure sores. Eur J Clin Nutr. (1988) 42:321–8. 3396523

[109] Dore-Duffy P Peterson M Catalanotto F Marlow S Ho SY Ostrom M. Zinc profiles in rheumatoid arthritis. Clin Exp Rheumatol. (1990) 8:541–6. 2289324

[110] Bideci A Camurdan MO Cinaz P Dursun H Demirel F. Serum zinc, insulin-like growth factor-I and insulin-like growth factor binding protein-3 levels in children with type 1 diabetes mellitus. J Pediatr Endocrinol Metab. (2005) 18:1007–11. 10.1515/JPEM.2005.18.10.1007 16355814

[111] Sanna A Firinu D Zavattari P Valera P. Zinc status and autoimmunity: a systematic review and meta-analysis. Nutrients (2018) 10: E68. 10.3390/nu10010068 29324654 PMC5793296

[112] Souffriau J Libert C. Mechanistic insights into the protective impact of zinc on sepsis. Cytokine Growth Factor Rev. (2018) 39:92–101. 10.1016/j.cytogfr.2017.12.002 29279185

[113] Ganatra HA Varisco BM Harmon K Lahni P Opoka A Wong HR. Zinc supplementation leads to immune modulation and improved survival in a juvenile model of murine sepsis. Innate Immun. (2017) 23:67–76. 10.1177/1753425916677073 27821649 PMC8865134

[114] Wessels I Cousins RJ. Zinc dyshomeostasis during polymicrobial sepsis in mice involves zinc transporter Zip14 and can be overcome by zinc supplementation. Am J Physiol Gastrointest Liver Physiol. (2015) 309:G768–78. 10.1152/ajpgi.00179.2015 26272258 PMC4628964

[115] Banupriya N Bhat BV Benet BD Catherine C Sridhar MG Parija SC. Short term oral zinc supplementation among babies with neonatal sepsis for reducing mortality and improving outcome - A double-blind randomized controlled trial. Indian J Pediatr. (2018) 85:5–9. 10.1007/s12098-017-2444-8 28891027

[116] Banupriya N Vishnu Bhat B Benet BD Sridhar MG Parija SC. Efficacy of zinc supplementation on serum calprotectin, inflammatory cytokines and outcome in neonatal sepsis - a randomized controlled trial. J Matern Fetal Neonatal Med. (2017) 30:1627–31. 10.1080/14767058.2016.1220524 27491377

[117] Alker W Haase H. Zinc and sepsis. Nutrients (2018) 10:976. 10.3390/nu10080976 30060473 PMC6115943

[118] Calder PC. Omega-3 fatty acids and inflammatory processes: from molecules to man. Biochem Soc Trans. (2017) 45:1105–15. 10.1042/BST20160474 28900017

[119] Fritsche K. Fatty acids as modulators of the immune response. Annu Rev Nutr. (2006) 26:45–73. 10.1146/annurev.nutr.25.050304.092610 16848700

[120] Galli C Calder PC. Effects of fat and fatty acid intake on inflammatory and immune responses: a critical review. Ann Nutr Metab. (2009) 55:123–39. 10.1159/000228999 19752539

[121] Kim W Khan NA McMurray DN Prior IA Wang N Chapkin RS. Regulatory activity of polyunsaturated fatty acids in T-cell signaling. Prog Lipid Res. (2010) 49:250–61. 10.1016/j.plipres.2010.01.002 20176053 PMC2872685

[122] Shaikh SR Edidin M. Polyunsaturated fatty acids, membrane organization, T cells, and antigen presentation. Am J Clin Nutr. (2006) 84:1277–89. 10.1093/ajcn/84.6.1277 17158407

[123] Whelan J Gowdy KM Shaikh SR. N-3 polyunsaturated fatty acids modulate B cell activity in pre-clinical models: implications for the immune response to infections. Eur J Pharmacol. (2016) 785:10–7. 10.1016/j.ejphar.2015.03.100 26022530 PMC4662641

[124] Zhao Y Joshi-Barve S Barve S Chen LH. Eicosapentaenoic acid prevents LPS-induced TNF-alpha expression by preventing NF-kappaB activation. J Am Coll Nutr. (2004) 23:71–8. 10.1080/07315724.2004.10719345 14963056

[125] Mullen A Loscher CE Roche HM. Anti-inflammatory effects of EPA and DHA are dependent upon time and dose-response elements associated with LPS stimulation in THP-1-derived macrophages. J Nutr Biochem. (2010) 21:444–50. 10.1016/j.jnutbio.2009.02.008 19427777

[126] Serhan CN. Pro-resolving lipid mediators are leads for resolution physiology. Nature (2014) 510:92–101. 10.1038/nature13479 24899309 PMC4263681

[127] Serhan CN Chiang N Van Dyke TE. Resolving inflammation: dual anti-inflammatory and pro-resolution lipid mediators. Nat Rev Immunol. (2008) 8:349–61. 10.1038/nri2294 18437155 PMC2744593

[128] Schwab JM Chiang N Arita M Serhan CN. Resolvin E1 and protectin D1 activate inflammation-resolution programmes. Nature (2007) 447:869–74. 10.1038/nature05877 17568749 PMC2757086

[129] Hong S Gronert K Devchand PR Moussignac RL Serhan CN. Novel docosatrienes and 17S-resolvins generated from docosahexaenoic acid in murine brain, human blood, and glial cells. Autacoids in anti-inflammation. J Biol Chem. (2003) 278:14677–87. 10.1074/jbc.M300218200 12590139

[130] Arita M Yoshida M Hong S Tjonahen E Glickman JN Petasis NA. Resolvin E1, an endogenous lipid mediator derived from omega-3 eicosapentaenoic acid, protects against 2,4,6-trinitrobenzene sulfonic acid-induced colitis. Proc Natl Acad Sci USA. (2005) 102:7671–6. 10.1073/pnas.0409271102 15890784 PMC1103706

[131] Weylandt KH Kang JX Wiedenmann B Baumgart DC. Lipoxins and resolvins in inflammatory bowel disease. Inflamm Bowel Dis. (2007) 13:797–9. 10.1002/ibd.20109 17262807

[132] Marcon R Bento AF Dutra RC Bicca MA Leite DF Calixto JB. Maresin 1, a proresolving lipid mediator derived from omega-3 polyunsaturated fatty acids, exerts protective actions in murine models of colitis. J Immunol. (2013) 191:4288–98. 10.4049/jimmunol.1202743 24038091

[133] Fan YY Ly LH Barhoumi R McMurray DN Chapkin RS. Dietary docosahexaenoic acid suppresses T cell protein kinase C theta lipid raft recruitment and IL-2 production. J Immunol. (2004) 173:6151–60. 10.4049/jimmunol.173.10.6151 15528352

[134] Switzer KC Fan YY Wang N McMurray DN Chapkin RS. Dietary n-3 polyunsaturated fatty acids promote activation-induced cell death in Th1-polarized murine CD4+ T-cells. J Lipid Res. (2004) 45:1482–92. 10.1194/jlr.M400028-JLR200 15145980 PMC4469998

[135] Pompos LJ Fritsche KL. Antigen-driven murine CD4+ T lymphocyte proliferation and interleukin-2 production are diminished by dietary (n-3) polyunsaturated fatty acids. J Nutr. (2002) 132:3293–300. 10.1093/jn/132.11.3293 12421842

[136] Meydani SN Lichtenstein AH Cornwall S Meydani M Goldin BR Rasmussen H. Immunologic effects of national cholesterol education panel step-2 diets with and without fish-derived N-3 fatty acid enrichment. J Clin Invest. (1993) 92:105–13. 10.1172/JCI116537 8325975 PMC293543

[137] Kim W Fan YY Barhoumi R Smith R McMurray DN Chapkin RS. n-3 polyunsaturated fatty acids suppress the localization and activation of signaling proteins at the immunological synapse in murine CD4+ T cells by affecting lipid raft formation. J Immunol. (2008) 181:6236–43. 10.4049/jimmunol.181.9.6236 18941214 PMC2597670

[138] Yog R Barhoumi R McMurray DN Chapkin RS. n-3 polyunsaturated fatty acids suppress mitochondrial translocation to the immunologic synapse and modulate calcium signaling in T cells. J Immunol. (2010) 184:5865–73. 10.4049/jimmunol.0904102 20393134 PMC4422833

[139] Fan YY Fuentes NR Hou TY Barhoumi R Li XC Deutz NEP. Remodelling of primary human CD4+ T cell plasma membrane order by n-3 PUFA. Br J Nutr. (2018) 119:163–75. 10.1017/S0007114517003385 29249211 PMC5927572

[140] Hou TY McMurray DN Chapkin RS. Omega-3 fatty acids, lipid rafts, and T cell signaling. Eur J Pharmacol. (2016) 785:2–9. 10.1016/j.ejphar.2015.03.091 26001374 PMC4654711

[141] Miguel L Owen DM Lim C Liebig C Evans J Magee AI. Primary human CD4+ T cells have diverse levels of membrane lipid order that correlate with their function. J Immunol. (2011) 186:3505–16. 10.4049/jimmunol.1002980 21307290

[142] Shaikh SR Jolly CA Chapkin RS. n-3 Polyunsaturated fatty acids exert immunomodulatory effects on lymphocytes by targeting plasma membrane molecular organization. Mol Aspects Med. (2012) 33:46–54. 10.1016/j.mam.2011.10.002 22020145 PMC3246093

[143] Kim W Barhoumi R McMurray DN Chapkin RS. Dietary fish oil and DHA down-regulate antigen-activated CD4+ T-cells while promoting the formation of liquid-ordered mesodomains. Br J Nutr. (2014) 111:254–60. 10.1017/S0007114513002444 23962659 PMC4327854

[144] Monk JM Hou TY Turk HF McMurray DN Chapkin RS. n3 PUFAs reduce mouse CD4+ T-cell *ex vivo* polarization into Th17 cells. J Nutr. (2013) 143:1501–8. 10.3945/jn.113.178178 23864512 PMC3743278

[145] Monk JM Hou TY Turk HF Weeks B Wu C McMurray DN. Dietary n-3 polyunsaturated fatty acids (PUFA) decrease obesity-associated Th17 cell-mediated inflammation during colitis. PLoS ONE (2012) 7:e49739. 10.1371/journal.pone.0049739 23166761 PMC3500317

[146] Zhang P Smith R Chapkin RS McMurray DN. Dietary (n-3) polyunsaturated fatty acids modulate murine Th1/Th2 balance toward the Th2 pole by suppression of Th1 development. J Nutr. (2005) 135:1745–51. 10.1093/jn/135.7.1745 15987859

[147] Bi X Li F Liu S Jin Y Zhang X Yang T. omega-3 polyunsaturated fatty acids ameliorate type 1 diabetes and autoimmunity. J Clin Invest. (2017) 127:1757–71. 10.1172/JCI87388 28375156 PMC5409789

[148] Fujikawa M Yamashita N Yamazaki K Sugiyama E Suzuki H Hamazaki T. Eicosapentaenoic acid inhibits antigen-presenting cell function of murine splenocytes. Immunology (1992) 75:330–5. 1551695 PMC1384715

[149] Hughes DA Pinder AC Piper Z Johnson IT Lund EK. Fish oil supplementation inhibits the expression of major histocompatibility complex class II molecules and adhesion molecules on human monocytes. Am J Clin Nutr. (1996) 63:267–72. 10.1093/ajcn/63.2.267 8561070

[150] Hughes DA Southon S Pinder AC. (n-3) Polyunsaturated fatty acids modulate the expression of functionally associated molecules on human monocytes *in vitro*. J Nutr. (1996) 126:603–10. 10.1093/jn/126.3.603 8598544

[151] Sanderson P MacPherson GG Jenkins CH Calder PC. Dietary fish oil diminishes the antigen presentation activity of rat dendritic cells. J Leukoc Biol. (1997) 62:771–7. 10.1002/jlb.62.6.771 9400818

[152] Zapata-Gonzalez F Rueda F Petriz J Domingo P Villarroya F Diaz-Delfin J. Human dendritic cell activities are modulated by the omega-3 fatty acid, docosahexaenoic acid, mainly through PPAR(gamma):RXR heterodimers: comparison with other polyunsaturated fatty acids. J Leukoc Biol. (2008) 84:1172–82. 10.1189/jlb.1007688 18632990

[153] Teague H Rockett BD Harris M Brown DA Shaikh SR. Dendritic cell activation, phagocytosis and CD69 expression on cognate T cells are suppressed by n-3 long-chain polyunsaturated fatty acids. Immunology (2013) 139:386–94. 10.1111/imm.12088 23373457 PMC3701185

[154] Rockett BD Melton M Harris M Bridges LC Shaikh SR. Fish oil disrupts MHC class II lateral organization on the B-cell side of the immunological synapse independent of B-T cell adhesion. J Nutr Biochem. (2013) 24:1810–6. 10.1016/j.jnutbio.2013.02.013 23791516 PMC3785547

[155] Rockett BD Teague H Harris M Melton M Williams J Wassall SR. Fish oil increases raft size and membrane order of B cells accompanied by differential effects on function. J Lipid Res. (2012) 53:674–85. 10.1194/jlr.M021782 22315394 PMC3307644

[156] Gurzell EA Teague H Duriancik D Clinthorne J Harris M Shaikh SR Marine fish oils are not equivalent with respect to B-cell membrane organization and activation. J Nutr Biochem. (2015) 26:369–77. 10.1016/j.jnutbio.2014.11.005 25616447 PMC4391512

[157] Gurzell EA Teague H Harris M Clinthorne J Shaikh SR Fenton JI. DHA-enriched fish oil targets B cell lipid microdomains and enhances *ex vivo* and *in vivo* B cell function. J Leukoc Biol. (2013) 93:463–70. 10.1189/jlb.0812394 23180828 PMC3597837

[158] Teague H Harris M Fenton J Lallemand P Shewchuk BM Shaikh SR. Eicosapentaenoic and docosahexaenoic acid ethyl esters differentially enhance B-cell activity in murine obesity. J Lipid Res. (2014) 55:1420–33. 10.1194/jlr.M049809 24837990 PMC4076074

[159] Calder PC. Marine omega-3 fatty acids and inflammatory processes: effects, mechanisms and clinical relevance. Biochim Biophys Acta (2015) 1851:469–84. 10.1016/j.bbalip.2014.08.010 25149823

[160] Calder PC. Fatty acids and immune function: relevance to inflammatory bowel diseases. Int Rev Immunol. (2009) 28:506–34. 10.3109/08830180903197480 19954361

[161] Fernandes G Bhattacharya A Rahman M Zaman K Banu J. Effects of n-3 fatty acids on autoimmunity and osteoporosis. Front Biosci. (2008) 13:4015–20. 10.2741/2989 18508495

[162] Miles EA Calder PC. Influence of marine n-3 polyunsaturated fatty acids on immune function and a systematic review of their effects on clinical outcomes in rheumatoid arthritis. Br J Nutr. (2012) 107(Suppl. 2):S171–84. 10.1017/S0007114512001560 22591891

[163] Hudert CA Weylandt KH Lu Y Wang J Hong S Dignass A. Transgenic mice rich in endogenous omega-3 fatty acids are protected from colitis. Proc Natl Acad Sci USA. (2006) 103:11276–81. 10.1073/pnas.0601280103 16847262 PMC1544078

[164] Ishida T Yoshida M Arita M Nishitani Y Nishiumi S Masuda A. Resolvin E1, an endogenous lipid mediator derived from eicosapentaenoic acid, prevents dextran sulfate sodium-induced colitis. Inflamm Bowel Dis. (2010) 16:87–95. 10.1002/ibd.21029 19572372 PMC3070396

[165] Matsunaga H Hokari R Kurihara C Okada Y Takebayashi K Okudaira K. Omega-3 fatty acids exacerbate DSS-induced colitis through decreased adiponectin in colonic subepithelial myofibroblasts. Inflamm Bowel Dis. (2008) 14:1348–57. 10.1002/ibd.20491 18484673

[166] Woodworth HL McCaskey SJ Duriancik DM Clinthorne JF Langohr IM Gardner EM. Dietary fish oil alters T lymphocyte cell populations and exacerbates disease in a mouse model of inflammatory colitis. Cancer Res. (2010) 70:7960–9. 10.1158/0008-5472.CAN-10-1396 20798218

[167] Belluzzi A Brignola C Campieri M Pera A Boschi S Miglioli M. Effect of an enteric-coated fish-oil preparation on relapses in Crohn's disease. N Engl J Med. (1996) 334:1557–60. 10.1056/NEJM199606133342401 8628335

[168] Seidner DL Lashner BA Brzezinski A Banks PL Goldblum J Fiocchi C. An oral supplement enriched with fish oil, soluble fiber, and antioxidants for corticosteroid sparing in ulcerative colitis: a randomized, controlled trial. Clin Gastroenterol Hepatol. (2005) 3:358–69. 10.1016/S1542-3565(04)00672-X 15822041

[169] Lev-Tzion R Griffiths AM Leder O Turner D. Omega 3 fatty acids (fish oil) for maintenance of remission in Crohn's disease. Cochrane Database Syst Rev. (2014) CD006320. 10.1002/14651858.CD006320.pub4 24585498 PMC8988157

[170] Turner D Shah PS Steinhart AH Zlotkin S Griffiths AM. Maintenance of remission in inflammatory bowel disease using omega-3 fatty acids (fish oil): a systematic review and meta-analyses. Inflamm Bowel Dis. (2011) 17:336–45. 10.1002/ibd.21374 20564531

[171] Arm JP Horton CE Mencia-Huerta JM House F Eiser NM Clark TJ. Effect of dietary supplementation with fish oil lipids on mild asthma. Thorax (1988) 43:84–92. 10.1136/thx.43.2.84 3353893 PMC1020747

[172] Broughton KS Johnson CS Pace BK Liebman M Kleppinger KM. Reduced asthma symptoms with n-3 fatty acid ingestion are related to 5-series leukotriene production. Am J Clin Nutr. (1997) 65:1011–7. 10.1093/ajcn/65.4.1011 9094887

[173] Surette ME Stull D Lindemann J. The impact of a medical food containing gammalinolenic and eicosapentaenoic acids on asthma management and the quality of life of adult asthma patients. Curr Med Res Opin. (2008) 24:559–67. 10.1185/030079908X273011 18194593

[174] Hodge L Salome CM Hughes JM Liu-Brennan D Rimmer J Allman M. Effect of dietary intake of omega-3 and omega-6 fatty acids on severity of asthma in children. Eur Respir J. (1998) 11:361–5. 10.1183/09031936.98.11020361 9551739

[175] Schubert R Kitz R Beermann C Rose MA Lieb A Sommerer PC. Effect of n-3 polyunsaturated fatty acids in asthma after low-dose allergen challenge. Int Arch Allergy Immunol. (2009) 148:321–9. 10.1159/000170386 19001792

[176] Kumar A Mastana SS Lindley MR. n-3 Fatty acids and asthma. Nutr Res Rev. (2016) 29:1–16. 10.1017/S0954422415000116 26809946

[177] Anandan C Nurmatov U Sheikh A. Omega 3 and 6 oils for primary prevention of allergic disease: systematic review and meta-analysis. Allergy (2009) 64:840–8. 10.1111/j.1398-9995.2009.02042.x 19392990

[178] Schachter HM Reisman J Tran K Dales B Kourad K Barnes D. Health effects of omega-3 fatty acids on asthma. In: Evidence Reports/Technology Assessments, No. 91 (2004). p. 1–7. 15133885 PMC4781132

[179] Goldberg RJ Katz J. A meta-analysis of the analgesic effects of omega-3 polyunsaturated fatty acid supplementation for inflammatory joint pain. Pain (2007) 129:210–23. 10.1016/j.pain.2007.01.020 17335973

[180] Gioxari A Kaliora AC Marantidou F Panagiotakos DP. Intake of omega-3 polyunsaturated fatty acids in patients with rheumatoid arthritis: a systematic review and meta-analysis. Nutrition (2018) 45:114–24 e4. 10.1016/j.nut.2017.06.023 28965775

[181] Norris JM Yin X Lamb MM Barriga K Seifert J Hoffman M. Omega-3 polyunsaturated fatty acid intake and islet autoimmunity in children at increased risk for type 1 diabetes. JAMA (2007) 298:1420–8. 10.1001/jama.298.12.1420 17895458

[182] Stene LC Joner G Norwegian Childhood Diabetes Study G. Use of cod liver oil during the first year of life is associated with lower risk of childhood-onset type 1 diabetes: a large, population-based, case-control study. Am J Clin Nutr. (2003) 78:1128–34. 10.1093/ajcn/78.6.1128 14668274

[183] FAO/WHO Evaluation of Health and Nutritional Properties of Probiotics in Food Including Powerder Milk and Live Lactic Acid Bacteria. Food and Agriculture Organization of the United Nations and World Health Organization Expert Consultation Report (2001). p. 1–34.

[184] Hill C Guarner F Reid G Gibson GR Merenstein DJ Pot B. Expert consensus document. The International Scientific Association for Probiotics and Prebiotics consensus statement on the scope and appropriate use of the term probiotic. Nat Rev Gastroenterol Hepatol. (2014) 11:506–14. 10.1038/nrgastro.2014.66 24912386

[185] Galdeano CM Perdigon G. Role of viability of probiotic strains in their persistence in the gut and in mucosal immune stimulation. J Appl Microbiol. (2004) 97:673–81. 10.1111/j.1365-2672.2004.02353.x 15357716

[186] Thomas CM Versalovic J. Probiotics-host communication: modulation of signaling pathways in the intestine. Gut Microbes (2010) 1:1–16. 10.4161/gmic.1.3.11712 20672012 PMC2909492

[187] Macpherson AJ Uhr T. Induction of protective IgA by intestinal dendritic cells carrying commensal bacteria. Science (2004) 303:1662–5. 10.1126/science.1091334 15016999

[188] Muscettola M Massai L Tanganelli C Grasso G. Effects of lactobacilli on interferon production in young and aged mice. Ann N Y Acad Sci. (1994) 717:226–32. 10.1111/j.1749-6632.1994.tb12092.x 8030839

[189] Baba N Samson S Bourdet-Sicard R Rubio M Sarfati M. Selected commensal-related bacteria and Toll-like receptor 3 agonist combinatorial codes synergistically induce interleukin-12 production by dendritic cells to trigger a T helper type 1 polarizing programme. Immunology (2009) 128:e523–31. 10.1111/j.1365-2567.2008.03022.x 19740313 PMC2753912

[190] Rescigno M Urbano M Valzasina B Francolini M Rotta G Bonasio R. Dendritic cells express tight junction proteins and penetrate gut epithelial monolayers to sample bacteria. Nat Immunol. (2001) 2:361–7. 10.1038/86373 11276208

[191] Vientos-Plotts AI Ericsson AC Rindt H Reinero CR. Oral probiotics alter healthy feline respiratory microbiota. Front Microbiol. (2017) 8:1287. 10.3389/fmicb.2017.01287 28744273 PMC5504723

[192] Davidson LE Fiorino AM Snydman DR Hibberd PL. Lactobacillus GG as an immune adjuvant for live-attenuated influenza vaccine in healthy adults: a randomized double-blind placebo-controlled trial. Eur J Clin Nutr. (2011) 65:501–7. 10.1038/ejcn.2010.289 21285968 PMC3071884

[193] Elmadfa I Klein P Meyer AL. Immune-stimulating effects of lactic acid bacteria *in vivo* and *in vitro*. Proc Nutr Soc. (2010) 69:416–20. 10.1017/S0029665110001710 20550748

[194] Vidal K Benyacoub J Moser M Sanchez-Garcia J Serrant P Segura-Roggero I. Effect of *Lactobacillus paracasei* NCC2461 on antigen-specific T-cell mediated immune responses in aged mice. Rejuvenation Res. (2008) 11:957–64. 10.1089/rej.2008.0780 18922048

[195] Borchers AT Keen CL Gershwin ME. The influence of yogurt/Lactobacillus on the innate and acquired immune response. Clin Rev Allergy Immunol. (2002) 22:207–30. 10.1007/s12016-002-0009-7 12043382

[196] Delcenserie V Martel D Lamoureux M Amiot J Boutin Y Roy D. Immunomodulatory effects of probiotics in the intestinal tract. Curr Issues Mol Biol. (2008) 10:37–54. 18525105

[197] Arunachalam K Gill HS Chandra RK. Enhancement of natural immune function by dietary consumption of Bifidobacterium lactis (HN019). Eur J Clin Nutr. (2000) 54:263–7. 10.1038/sj.ejcn.1600938 10713750

[198] Kekkonen RA Lummela N Karjalainen H Latvala S Tynkkynen S Jarvenpaa S. Probiotic intervention has strain-specific anti-inflammatory effects in healthy adults. World J Gastroenterol. (2008) 14:2029–36. 10.3748/wjg.14.2029 18395902 PMC2701523

[199] Spanhaak S Havenaar R Schaafsma G. The effect of consumption of milk fermented by *Lactobacillus casei* strain Shirota on the intestinal microflora and immune parameters in humans. Eur J Clin Nutr. (1998) 52:899–907. 10.1038/sj.ejcn.1600663 9881885

[200] Koch S Nusrat A. The life and death of epithelia during inflammation: lessons learned from the gut. Annu Rev Pathol. (2012) 7:35–60. 10.1146/annurev-pathol-011811-120905 21838548

[201] Kang HJ Im SH. Probiotics as an immune modulator. J Nutr Sci Vitaminol. (2015) 61(Suppl.):S103–5. 10.3177/jnsv.61.S103 26598815

[202] Tobita K Yanaka H Otani H. *Lactobacillus crispatus* KT-11 enhances intestinal immune functions in C3H/HeN mice. J Nutr Sci Vitaminol. (2010) 56:441–5. 10.3177/jnsv.56.441 21422714

[203] Ya T Zhang Q Chu F Merritt J Bilige M Sun T. Immunological evaluation of *Lactobacillus casei* Zhang: a newly isolated strain from koumiss in Inner Mongolia, China. BMC Immunol. (2008) 9:68. 10.1186/1471-2172-9-68 19019236 PMC2596084

[204] Yang HY Liu SL Ibrahim SA Zhao L Jiang JL Sun WF. Oral administration of live Bifidobacterium substrains isolated from healthy centenarians enhanced immune function in BALB/c mice. Nutr Res. (2009) 29:281–9. 10.1016/j.nutres.2009.03.010 19410981

[205] Lee Y Lee TS. Enhancement in *ex vivo* phagocytic capacity of peritoneal leukocytes in mice by oral delivery of various lactic-acid-producing bacteria. Curr Microbiol. (2005) 50:24–7. 10.1007/s00284-004-4377-5 15696260

[206] Tsai YT Cheng PC Fan CK Pan TM. Time-dependent persistence of enhanced immune response by a potential probiotic strain *Lactobacillus paracasei* subsp. paracasei NTU 101. Int J Food Microbiol. (2008) 128:219–25. 10.1016/j.ijfoodmicro.2008.08.009 18809220

[207] Gill HS Rutherfurd KJ Cross ML. Dietary probiotic supplementation enhances natural killer cell activity in the elderly: an investigation of age-related immunological changes. J Clin Immunol. (2001) 21:264–71. 10.1023/A:1010979225018 11506196

[208] Sheih YH Chiang BL Wang LH Liao CK Gill HS. Systemic immunity-enhancing effects in healthy subjects following dietary consumption of the lactic acid bacterium *Lactobacillus rhamnosus* HN001. J Am Coll Nutr. (2001) 20:149–56. 10.1080/07315724.2001.10719027 11349938

[209] Parra MD Martinez de Morentin BE Cobo JM Mateos A Martinez JA. Daily ingestion of fermented milk containing Lactobacillus casei DN114001 improves innate-defense capacity in healthy middle-aged people. J Physiol Biochem. (2004) 60:85–91. 10.1007/BF03168444 15457926

[210] Fang H Elina T Heikki A Seppo S. Modulation of humoral immune response through probiotic intake. FEMS Immunol Med Microbiol. (2000) 29:47–52. 10.1111/j.1574-695X.2000.tb01504.x 10967260

[211] Pelto L Isolauri E Lilius EM Nuutila J Salminen S. Probiotic bacteria down-regulate the milk-induced inflammatory response in milk-hypersensitive subjects but have an immunostimulatory effect in healthy subjects. Clin Exp Allergy (1998) 28:1474–9. 10.1046/j.1365-2222.1998.00449.x 10024217

[212] Barberi C Campana S De Pasquale C Rabbani Khorasgani M Ferlazzo G Bonaccorsi I. T cell polarizing properties of probiotic bacteria. Immunol Lett. (2015) 168:337–42. 10.1016/j.imlet.2015.11.005 26554608

[213] Dwivedi M Kumar P Laddha NC Kemp EH. Induction of regulatory T cells: a role for probiotics and prebiotics to suppress autoimmunity. Autoimmun Rev. (2016) 15:379–92. 10.1016/j.autrev.2016.01.002 26774011

[214] Fu YR Yi ZJ Pei JL Guan S. Effects of *Bifidobacterium bifidum* on adaptive immune senescence in aging mice. Microbiol Immunol. (2010) 54:578–83. 10.1111/j.1348-0421.2010.00255.x 21118295

[215] Goodwin K Viboud C Simonsen L. Antibody response to influenza vaccination in the elderly: a quantitative review. Vaccine (2006) 24:1159–69. 10.1016/j.vaccine.2005.08.105 16213065

[216] Boge T Remigy M Vaudaine S Tanguy J Bourdet-Sicard R van der Werf S. A probiotic fermented dairy drink improves antibody response to influenza vaccination in the elderly in two randomised controlled trials. Vaccine (2009) 27:5677–84. 10.1016/j.vaccine.2009.06.094 19615959

[217] Fukushima Y Miyaguchi S Yamano T Kaburagi T Iino H Ushida K. Improvement of nutritional status and incidence of infection in hospitalised, enterally fed elderly by feeding of fermented milk containing probiotic *Lactobacillus johnsonii* La1 (NCC533). Br J Nutr. (2007) 98:969–77. 10.1017/S0007114507764723 17617944

[218] Guillemard E Tondu F Lacoin F Schrezenmeir J. Consumption of a fermented dairy product containing the probiotic *Lactobacillus casei* DN-114001 reduces the duration of respiratory infections in the elderly in a randomised controlled trial. Br J Nutr. (2010) 103:58–68. 10.1017/S0007114509991395 19747410

[219] Turchet P Laurenzano M Auboiron S Antoine JM. Effect of fermented milk containing the probiotic *Lactobacillus casei* DN-114001 on winter infections in free-living elderly subjects: a randomised, controlled pilot study. J Nutr Health Aging (2003) 7:75–7. 12679825

[220] Makino S Ikegami S Kume A Horiuchi H Sasaki H Orii N. Reducing the risk of infection in the elderly by dietary intake of yoghurt fermented with *Lactobacillus delbrueckii* ssp. bulgaricus OLL1073R-1. Br J Nutr. (2010) 104:998–1006. 10.1017/S000711451000173X 20487575

[221] Mane J Pedrosa E Loren V Gassull MA Espadaler J Cune J. A mixture of *Lactobacillus plantarum* CECT 7315 and CECT 7316 enhances systemic immunity in elderly subjects. A dose-response, double-blind, placebo-controlled, randomized pilot trial. Nutr Hosp. (2011) 26:228–35. 21519752

[222] Ivory K Chambers SJ Pin C Prieto E Arques JL Nicoletti C. Oral delivery of *Lactobacillus casei* Shirota modifies allergen-induced immune responses in allergic rhinitis. Clin Exp Allergy (2008) 38:1282–9. 10.1111/j.1365-2222.2008.03025.x 18510694

[223] Ivory K Wilson AM Sankaran P Westwood M McCarville J Brockwell C. Oral delivery of a probiotic induced changes at the nasal mucosa of seasonal allergic rhinitis subjects after local allergen challenge: a randomised clinical trial. PLoS ONE (2013) 8:e78650. 10.1371/journal.pone.0078650 24260122 PMC3829814

[224] Ghadimi D Folster-Holst R de Vrese M Winkler P Heller KJ Schrezenmeir J. Effects of probiotic bacteria and their genomic DNA on TH1/TH2-cytokine production by peripheral blood mononuclear cells (PBMCs) of healthy and allergic subjects. Immunobiology (2008) 213:677–92. 10.1016/j.imbio.2008.02.001 18950596

[225] Pae M Wu D. Immunomodulating effects of epigallocatechin-3-gallate from green tea: mechanisms and applications. Food Funct. (2013) 4:1287–303. 10.1039/c3fo60076a 23835657

[226] Dona M Dell'Aica I Calabrese F Benelli R Morini M Albini A. Neutrophil restraint by green tea: inhibition of inflammation, associated angiogenesis, and pulmonary fibrosis. J Immunol. (2003) 170:4335–41. 10.4049/jimmunol.170.8.4335 12682270

[227] Takano K Nakaima K Nitta M Shibata F Nakagawa H. Inhibitory effect of (-)-epigallocatechin 3-gallate, a polyphenol of green tea, on neutrophil chemotaxis *in vitro* and *in vivo*. J Agric Food Chem. (2004) 52:4571–6. 10.1021/jf0355194 15237969

[228] Chan KH Chan SC Yeung SC Man RY Ip MS Mak JC. Inhibitory effect of Chinese green tea on cigarette smoke-induced up-regulation of airway neutrophil elastase and matrix metalloproteinase-12 via antioxidant activity. Free Radic Res. (2012) 46:1123–9. 10.3109/10715762.2012.692786 22574903

[229] Melgarejo E Medina MA Sanchez-Jimenez F Urdiales JL. Epigallocatechin gallate reduces human monocyte mobility and adhesion *in vitro*. Br J Pharmacol. (2009) 158:1705–12. 10.1111/j.1476-5381.2009.00452.x 19912233 PMC2801211

[230] Murakami A Takahashi D Hagihara K Koshimizu K Ohigashi H. Combinatorial effects of nonsteroidal anti-inflammatory drugs and food constituents on production of prostaglandin E2 and tumor necrosis factor-alpha in RAW264.7 murine macrophages. Biosci Biotechnol Biochem. (2003) 67:1056–62. 10.1271/bbb.67.1056 12834283

[231] Park JW Choi YJ Suh SI Kwon TK. Involvement of ERK and protein tyrosine phosphatase signaling pathways in EGCG-induced cyclooxygenase-2 expression in Raw 264.7 cells. Biochem Biophys Res Commun. (2001) 286:721–5. 10.1006/bbrc.2001.5415 11520057

[232] Matsunaga K Klein TW Friedman H Yamamoto Y. *Legionella pneumophila* replication in macrophages inhibited by selective immunomodulatory effects on cytokine formation by epigallocatechin gallate, a major form of tea catechins. Infect Immun. (2001) 69:3947–53. 10.1128/IAI.69.6.3947-3953.2001 11349063 PMC98432

[233] Pae M Ren Z Meydani M Shang F Smith D Meydani SN. Dietary supplementation with high dose of epigallocatechin-3-gallate promotes inflammatory response in mice. J Nutr Biochem. (2012) 23:526–31. 10.1016/j.jnutbio.2011.02.006 21684134

[234] Ahn SC Kim GY Kim JH Baik SW Han MK Lee HJ. Epigallocatechin-3-gallate, constituent of green tea, suppresses the LPS-induced phenotypic and functional maturation of murine dendritic cells through inhibition of mitogen-activated protein kinases and NF-kappaB. Biochem Biophys Res Commun. (2004) 313:148–55. 10.1016/j.bbrc.2003.11.108 14672711

[235] Yoneyama S Kawai K Tsuno NH Okaji Y Asakage M Tsuchiya T. Epigallocatechin gallate affects human dendritic cell differentiation and maturation. J Allergy Clin Immunol. (2008) 121:209–14. 10.1016/j.jaci.2007.08.026 17935769

[236] Hu ZQ Toda M Okubo S Hara Y Shimamura T. Mitogenic activity of (-)epigallocatechin gallate on B-cells and investigation of its structure-function relationship. Int J Immunopharmacol. (1992) 14:1399–407. 10.1016/0192-0561(92)90011-9 1464471

[237] Liu D Li P Song S Liu Y Wang Q Chang Y. Pro-apoptotic effect of epigallo-catechin-3-gallate on B lymphocytes through regulating BAFF/PI3K/Akt/mTOR signaling in rats with collagen-induced arthritis. Eur J Pharmacol. (2012) 690:214–25. 10.1016/j.ejphar.2012.06.026 22760071

[238] Wu D Guo Z Ren Z Guo W Meydani SN. Green tea EGCG suppresses T cell proliferation through impairment of IL-2/IL-2 receptor signaling. Free Radic Biol Med. (2009) 47:636–43. 10.1016/j.freeradbiomed.2009.06.001 19501156

[239] Pae M Ren Z Meydani M Shang F Meydani SN Wu D. Epigallocatechin-3-gallate directly suppresses T cell proliferation through impaired IL-2 utilization and cell cycle progression. J Nutr. (2010) 140:1509–15. 10.3945/jn.110.124743 20534878

[240] Kim JY Kina T Iwanaga Y Noguchi H Matsumura K Hyon SH. Tea polyphenol inhibits allostimulation in mixed lymphocyte culture. Cell Transplant. (2007) 16:75–83. 10.3727/000000007783464515 17436857

[241] Watson JL Vicario M Wang A Moreto M McKay DM. Immune cell activation and subsequent epithelial dysfunction by Staphylococcus enterotoxin B is attenuated by the green tea polyphenol (-)-epigallocatechin gallate. Cell Immunol. (2005) 237:7–16. 10.1016/j.cellimm.2005.08.030 16213476

[242] Wang J Pae M Meydani SN Wu D. Green tea epigallocatechin-3-gallate modulates differentiation of naive CD4(+) T cells into specific lineage effector cells. J Mol Med. (2013) 91:485–95. 10.1007/s00109-012-0964-2 23064699

[243] Wu CH Wu CF Huang HW Jao YC Yen GC. Naturally occurring flavonoids attenuate high glucose-induced expression of proinflammatory cytokines in human monocytic THP-1 cells. Mol Nutr Food Res. (2009) 53:984–95. 10.1002/mnfr.200800495 19557821

[244] Lyu SY Park WB. Production of cytokine and NO by RAW 264.7 macrophages and PBMC *in vitro* incubation with flavonoids. Arch Pharm Res. (2005) 28:573–81. 10.1007/BF02977761 15974445

[245] Wang J Pae M Meydani SN Wu D. Epigallocatechin-3-gallate inhibits expression of receptors for T cell regulatory cytokines and their downstream signaling in mouse CD4+ T cells. J Nutr. (2012) 142:566–71. 10.3945/jn.111.154419 22323768

[246] Shim JH Choi HS Pugliese A Lee SY Chae JI Choi BY. (-)-Epigallocatechin gallate regulates CD3-mediated T cell receptor signaling in leukemia through the inhibition of ZAP-70 kinase. J Biol Chem. (2008) 283:28370–9. 10.1074/jbc.M802200200 18687687 PMC2568917

[247] Nam S Smith DM Dou QP. Ester bond-containing tea polyphenols potently inhibit proteasome activity *in vitro* and *in vivo*. J Biol Chem. (2001) 276:13322–30. 10.1074/jbc.M004209200 11278274

[248] Wu D Wang J Pae M Meydani SN. Green tea EGCG, T cells, and T cell-mediated autoimmune diseases. Mol Aspects Med. (2012) 33:107–18. 10.1016/j.mam.2011.10.001 22020144

